# Aberrations of Chromosomes 1 and 16 in Breast Cancer: A Framework for Cooperation of Transcriptionally Dysregulated Genes

**DOI:** 10.3390/cancers13071585

**Published:** 2021-03-30

**Authors:** Anna Provvidenza Privitera, Vincenza Barresi, Daniele Filippo Condorelli

**Affiliations:** Department of Biomedical and Biotechnological Sciences, Section of Medical Biochemistry, University of Catania, Via S. Sofia 89-97, 95123 Catania, Italy; anna.privitera@phd.unict.it

**Keywords:** chromosome aberrations, cancer aneuploidy, gene copy number abnormalities, breast cancer, transcriptome, cancer genomics, BCL9, CDH1, gamma-secretase, cancer driver genes

## Abstract

**Simple Summary:**

Classical cytogenetic studies in breast cancer have identified frequent chromosomal aberrations that produce an increased gene copy number in chromosome 1q (1q-gain) and/or a decreased gene copy number in 16q (16q-loss). The understanding of the contribution of such copy number changes to the genesis and progression of cancer is of paramount importance for the design of cancer models and targeted therapies. We exploited molecular data provided by The Cancer Genome Atlas (TCGA) project in order to form different groups of breast cancers bearing 1q-gain and/or 16q-loss or devoid of such aberrations (1,16-chromogroups). An analysis of differential gene expression among 1,16-chromogroups guided the identification of transcriptionally dysregulated 1q and 16q genes. Pathway analysis revealed functional interactions that shed light on novel molecular targets for subtype-specific cancer therapy.

**Abstract:**

Derivative chromosome der(1;16), isochromosome 1q, and deleted 16q—producing arm-level 1q-gain and/or 16q-loss—are recurrent cytogenetic abnormalities in breast cancer, but their exact role in determining the malignant phenotype is still largely unknown. We exploited The Cancer Genome Atlas (TCGA) data to generate and analyze groups of breast invasive carcinomas, called 1,16-chromogroups, that are characterized by a pattern of arm-level somatic copy number aberrations congruent with known cytogenetic aberrations of chromosome 1 and 16. Substantial differences were found among 1,16-chromogroups in terms of other chromosomal aberrations, aneuploidy scores, transcriptomic data, single-point mutations, histotypes, and molecular subtypes. Breast cancers with a co-occurrence of 1q-gain and 16q-loss can be distinguished in a “low aneuploidy score” group, congruent to der(1;16), and a “high aneuploidy score” group, congruent to the co-occurrence of isochromosome 1q and deleted 16q. Another three groups are formed by cancers showing separately 1q-gain or 16q-loss or no aberrations of 1q and 16q. Transcriptome comparisons among the 1,16-chromogroups, integrated with functional pathway analysis, suggested the cooperation of overexpressed 1q genes and underexpressed 16q genes in the genesis of both ductal and lobular carcinomas, thus highlighting the putative role of genes encoding gamma-secretase subunits (APH1A, PSEN2, and NCSTN) and Wnt enhanceosome components (BCL9 and PYGO2) in 1q, and the glycoprotein E-cadherin (CDH1), the E3 ubiquitin-protein ligase WWP2, the deubiquitinating enzyme CYLD, and the transcription factor CBFB in 16q. The analysis of 1,16-chromogroups is a strategy with far-reaching implications for the selection of cancer cell models and novel experimental therapies.

## 1. Introduction

Derivative chromosome 1;16 (der(1;16)), isochromosome 1q (i(1q)), and deletion of 16q (del(16q))—producing arm-level 1q-gain and/or 16q-loss—are frequent recurrent cytogenetic abnormalities in breast cancer [1,2,3,4,5,6,7,8,9,10,11]. However, their exact role in determining the malignant phenotype is still largely unknown. Since no alterations of single genes have been revealed at or near the breakpoint junctions of those unbalanced chromosomal aberrations, there is a wide consensus that gene-dosage transcriptional effects are playing a role in tumorigenesis and cancer progression.

Derivative chromosome der(1;16) (q10;p10) is likely to exert a strong and specific driver effect in breast cancer, as suggested by the following results obtained in conventional and molecular cytogenetic studies [1,2,4,5,6,7,8,11]: (1) it is one of the most frequent recurrent chromosome aberration in breast cancer; (2) it has been frequently observed as the only karyotypic anomaly in breast cancer; (3) it is rather specific for breast cancer although it has also been reported in multiple myeloma, sarcomas, and Wilms’ tumor; and 4) it is assumed to be an early event in breast cancer progression and has been reported in a case of ductal carcinoma in situ.

The der(1;16) (q10;p10) is considered to be the consequence of an unbalanced centromere-close translocation t(1;16). In such case, a single chromosomal aberration event produces the simultaneous gain of 1q and loss of 16q. However, the presence of 1q-gain and 16q-loss can be also the result of two mechanistically distinct events, such as the generation of i(1q) and the deletion of 16q. Though the frequent association of 1q-gain and 16q-loss suggests a cooperation between those aberrations, it has been also reported that breast cancers can bear only 1q-gains or only 16-q losses [9,12].

In this work, we exploited the large amount of molecular cytogenetic data (single nucleotide polymorphism (SNP) array data) provided by The Cancer Genome Atlas (TCGA) study (http://cancergenome.nih.gov/ (accessed on 29 October 2019)) in order to generate groups of breast invasive carcinomas (here called 1,16-chromogroups) characterized by a pattern of arm-level somatic copy number aberrations congruent with the different cytogenetic abnormalities of chromosomes (chr) 1 and 16. We observed substantial differences among those 1,16-chromogroups in terms of other chromosomal aberrations, aneuploidy scores, transcriptomic data, and single-point mutation profiles. Such information, integrated with a comparative pathway analysis among different 1,16-chromogroups, suggests novel functional links among transcriptionally dysregulated genes in 1q and 16q in invasive ductal and lobular breast carcinomas.

## 2. Materials and Methods

### 2.1. RNA-Seq, Whole Exome Sequencing, Single Nucleotide Polymorphism (SNP) Arrays Data Collection

The TCGA data about breast invasive carcinoma (BRCA) were retrieved from the online data portal The Genomic Data Commons (GDC) (https://portal.gdc.cancer.gov accessed on 29 October 2019) [13,14]. Data from the following technologies were selected: (1) RNA-seq data (HTseq counts) from 1222 BRCA-TCGA samples and (2) whole exome sequencing (WES) data by using the available Mutation Annotation Format (MAF) file (variant calling by SomaticSniper algorithms [15]) from a total of 976 samples. Data regarding the cytogenetic features of BRCA were downloaded from cBioPortal for cancer genomics (https://www.cbioportal.org accessed on 29 October 2019) [16,17] for a total of 1084 samples (Affymetrix SNP 6.0 arrays).

### 2.2. Sample Selection and Gene Annotation

The RNA-seq data were filtered by selecting only 1072 primary tumors (TCGA-##-##-01A only primary tumor as sample type and only unique sample ID) samples and 99 normal mammary tissue samples (only TCGA-##-####-11A sample ID). SNP-array data were matched by using the corresponding sample ID to obtain a total of 1058 tumor samples with both RNA-seq and SNP-array data. Moreover, by using the corresponding case ID, we selected 946 samples with RNA-seq, SNP-array, and WES-seq data (See Table S1 for the sample IDs). The Stable Ensembl gene IDs (i.e., ENSG###) were matched with the corresponding gene name and additional annotation by using BioMart [18] and Genome Reference Consortium Human Build 38.p13 genome version (GRCh38.p13)(Ensembl Release 99; January 2020). All deprecated genes between GRCh37 and GRCh38 genome assemblies were not considered.

### 2.3. Normalization and Statistical Tools for RNA-Seq Data

The RNA-seq count data were pre-filtered to avoid background noise by removing low count genes (zero values in 70% of samples). The initial number of genes in RNA-seq count files was 60,483; after pre-filtering, we obtained 35,923 genes, and after removing deprecated genes, we obtained 35,903 genes. The RNA-seq BRCA counts were organized in 1,16-chromogroups, as described in the result section, and then normalized by using the trimmed mean of M-values (TMM) algorithm within the edgeR [19] and compcodeR [20] packages. The differential expression of transcripts between two cancer groups (a group with selected chromosome aberrations and a control (CTRL) group without 1,16 chromosome aberrations) or between a cancer group and the normal breast tissue group was calculated by the edgeR package. Results are expressed in linear fold-change or modified linear fold change. The Benjamini–Hochberg correction [21] was used to obtain the adjusted *p*-values (adjp) for multiple test comparisons. Differentially expressed genes (DEGs) of 1,16-chromogroups were classified in OverT (overexpressed transcript in comparison to CTRL), UnderT (Underexpressed Transcript in comparison to CTRL) and OverUpT (transcripts that are overexpressed in comparison to CTRL and upregulated in comparison to normal tissue) according to threshold values of linear fold changes and statistical significance (adjp), as described in the result section.

The densities of each class of transcripts in a chromosomal region (chromosomal arms of the entire genome or cytogenetic bands in a single chromosomal arm) were expressed as a normalized chromosomal distribution index (NCDI), calculated according to the following Formula (1) [22,23]:

(1) $$\mathrm{NCDI}\;\mathrm{of}\;\mathrm{OverT}\;\mathrm{or}\;\mathrm{UnderT}\;\mathrm{in}\;\mathrm{the}\;n-th\;\mathrm{chromosomal}\;\mathrm{region}=\frac{\frac{x_{n}}{X_{n}}*1}{\sum_{i=1}^{T}\frac{x_{i}}{X_{i}}}*100$$

where *x_n_* is the number of OverT or UnderT in the *n-th* chromosomal region, *X_n_* is the total number of transcripts encoded in the *n-th* chromosomal region, and T is the total number of chromosomal regions in the analyzed system (number of chromosomal arms in the entire genome or number of cytogenetic bands in the selected chromosomal arm).

The BRCA RNA-seq data normalized in fragments per kilobase per million reads mapped (FPKM) were retrieved from online data portal The Genomic Data Commons (GDC) (https://portal.gdc.cancer.gov accessed on 29 October 2019). The FPKM values of the same cohort of BRCA samples were converted in transcripts per million (TPM) according to standard conversion procedures.

### 2.4. Hierarchical Clustering

The hierarchical clustering algorithm [24] in the pheatmap [25] R package was used to group OverT and UnderT DEGs of the different 1,16 chromogroups. The rows correspond to genes, while columns correspond to 1,16-chromogroups. The values are the modified linear-fold-change (equal to “linear FC-1” if linear FC > 1 or to “linear FC + 1” if linear FC < 1). The row values were centered and scaled. The linkage agglomeration method was the unweighted pair group method with arithmetic mean (UP-GMA) applied to Euclidean distance.

### 2.5. Additional Bioinformatics and Statistics Tools

Pathway enrichment analysis was performed by the gene annotation and analysis resource Metascape [26]. Gene set enrichment analysis (GSEA) was performed by the publicly available software GSEAv4.1.0 (build:27, accessed on 7 September 2020), www.gsea-msigdb.org/gsea/index.jsp [27]. The WES-seq data were analyzed, summarized, and annotated by using the Maftools R Package [28]. The Venn diagrams were generated by the Interactive Venn [29]. The whole data organization was performed in R Studio (RStudio Team (2015) (RStudio: Integrated Development for R. RStudio, Inc., Boston, MA, USA, http://www.rstudio.com accessed on 29 October 2019)). The statistical analysis of the invasive lobular breast carcinomas and invasive ductal breast carcinomas was also performed using GraphPad Prism software version 8.0 (GraphPad Software, San Diego, CA, USA, www.graphpad.com accessed on 29 October 2019).

## 3. Results

A general schematic workflow of the analysis performed for the present report is shown in Figure S1. Briefly, after the cytogenomic characterization of 1,16-chromogroups, three main types of analysis of differential gene expression among 1,16-chromogroups were performed: (I) involving all BRCA samples, (II) restricted to ductal adenocarcinomas belonging to the LumA subtype, and (III) focused on the differences between ductal and lobular invasive adenocarcinomas.

### 3.1. Cytogenomics by SNP Array and 1,16-Chromogroups

**Figure 1:**
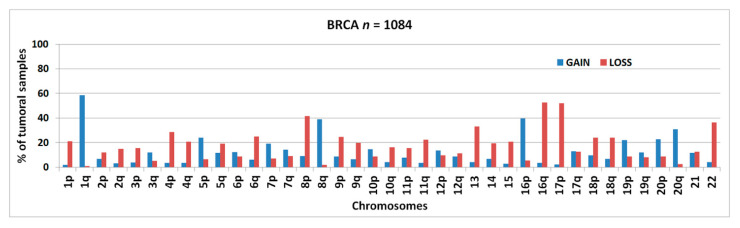
Percentage of samples bearing chromosomal arm-level gains or losses in 1084 breast invasive carcinoma (BRCA) samples.

Figure 1 shows the frequencies of chromosomal arm-level aberrations for the entire series of 1084 BRCA samples from TCGA. The gain of chr1q and the loss of chr16q were the most commonly detected abnormalities followed by 17p-loss, 16p-gain, 8p-loss, 8q-gain, 22-loss, 13q-loss, and 20q-gain.

In order to study the transcriptional effects of aberrations of chr1 and chr16, alone or in combination, we defined different subgroups of breast cancer samples according to the copy number status of those two chromosomes (1,16-chromogroups). As reported in the Mitelman Database of Chromosome Aberrations and Gene Fusions in Cancer (https://mitelmandatabase.isb-cgc.org/, accessed on 1 August 2020), two common cytogenetics abnormalities in breast cancer can underlie the gain of 1q: the derivative chromosome der(1;16) (q10;p10), formed by the short arm of chr16 and the long arm of chr1, as well as the isochromosome 1q, i(1q), formed by two long arms of chr1.

**Figure 2:**
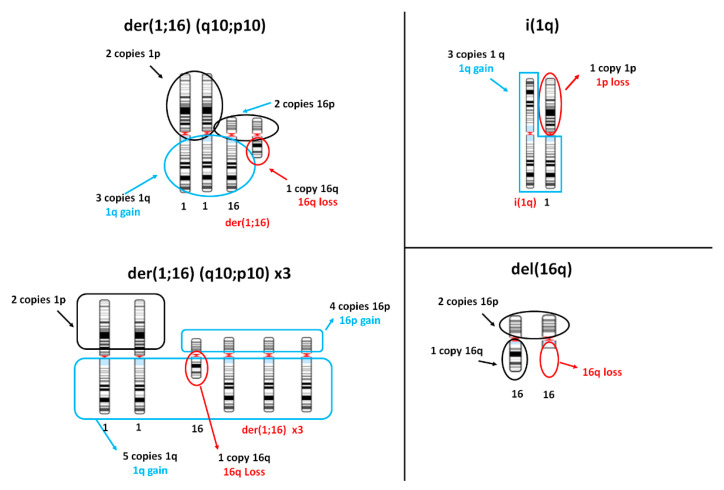
Schematic drawings of main cytogenetic aberrations in breast cancer and corresponding copy number changes. Left panel top: single copy of der(1;16) (q10;p10). Left panel bottom: 3 copies of der(1;16). Right panel top: isochromosome 1q (i(1q)), a chromosome formed by two long arms of chr1. Right panel bottom: deletion of long arm of chr16.

Considering the copy number changes frequently associated with the presence of der(1;16) (Figure 2, left panel; note that extra copies of der(1;16) are a common occurrence), we selected 178 BRCA samples composing group A, as reported in Table 1. Though we could not formally exclude the presence of other cytogenetic abnormalities leading to the same pattern of arm-level copy number changes in chr1 and chr16, we assumed that der(1;16) is highly enriched in tumors of group A, relying on the fact that it is one of the most common 1q-aberration shown in conventional cytogenetics studies in breast cancer [1,2,4,5,6,7,8,9,11]. Moreover, as reported below, group A showed distinctive properties from another group bearing concomitant 1q-gain and 16q-loss (group B1).

The i(1q) is another common aberration that produces a 1q-gain aberrations, and it was found to be as frequent as der(1;16). It is thought to be derived by an anomalous chromatid separation that leads to the generation of a chromosome formed by two 1q arms. Such aberration produces a 1q-gain associated with 1p-loss (Figure 2, right panel top). According to this pattern of copy number changes, we formed the so-called group B (*n* = 171). The groups A and B were disjoint because of the different condition established for Chr1p (disomic in group A or lost in group B). In group B, as a whole, we did not impose any criteria relative to the copy number status of chromosome 16 (Table 1), although a large fraction of samples of group B (67%) were found to bear a loss of 16q. However, we also formed a subset of group B, denominated subgroup B1, in which only samples showing a lost 16q and a gained or a disomic 16p were included (*n* = 101). Another subgroup of B, called B2, was formed by samples of group B bearing a disomic Chr16 (*n* = 18). Finally, a group C (*n* = 90) was formed by selecting samples bearing 1q-gain and disomic Chr16. Groups C and A were disjoint because of chr16 status, but group C was found to partially overlap with group B since it fully included subgroup B2.

In order to study the effects of the loss of chr16 not accompanied by aberrations of chr1, we also generated a group D (*n* = 75) characterized by normal Chr1 and 16q-loss. This group could be subdivided in two subgroups: the first one (*n* = 28), called subgroup D1, was formed by samples bearing 16q-loss and no arm-level copy number abnormalities in 16p, 1p, and 1q. Chr16 aberration in group D1 may correspond to the deletion of chr16q, del(16q), as observed in conventional cytogenetic studies (Figure 2). The second subgroup D (*n* = 47), called D2, was characterized by normal chr1, 16q-loss, and 16p-gain. No clear correspondence to reported conventional cytogenetic abnormalities could be identified for this subgroup. Subgroups D1 and D2 were disjoint because of the difference in chr16p (disomic in subgroup D1 and lost in subgroup D2).

In summary, group A and subgroup B1 contained BRCA samples bearing concomitant 1q-gain and 16q-loss, group C and subgroup B2 were formed by BRCA samples bearing 1q-gain in the absence of aberrations of chromosome 16, and Group D and its subgroups D1/D2 were formed by samples bearing 16q-loss in the absence of aberrations of chr1 (Table 1). Finally, we formed a group containing cancer samples not bearing any arm-level aberrations in chr1 and chr16. In the context of the present analysis, the latter group played a special role and was denominated control (CTRL) cancer group for this reason (Table 1). The basic assumption was that CTRL tumors follow a different evolutionary pathway towards malignancy. Therefore, the analysis of differential gene expression (other chromogroups versus CTRL group), as reported in the next sections, was used to generate lists of putative dosage-sensitive cancer driver genes associated with 1q-gain and/or 16q-loss.

**Figure 3:**
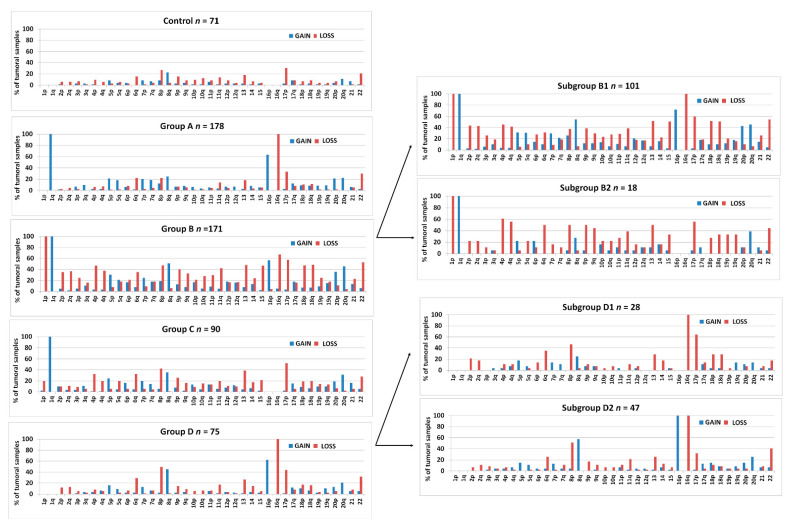
Bar graphs showing the percentage of samples bearing arm-level gains or losses in chromosomes 1–22 in the different 1,16-chromogroups (A, B, C, D, B1, B2, D1, D2, and control) of BRCA samples. The number of samples (*n*) in each group is reported in the corresponding graph.

Figure 3 shows the frequencies of arm-level aberrations in the above-defined 1,16-chromogroups. In accordance with the procedure followed for group formation, the main differences between the various groups involve chr1 and chr16. However, an increased frequency of 8q-gain in subgroups B1 and D2 and an increased frequency of losses of several other chromosomes in group B should be noted.

**Figure 4:**
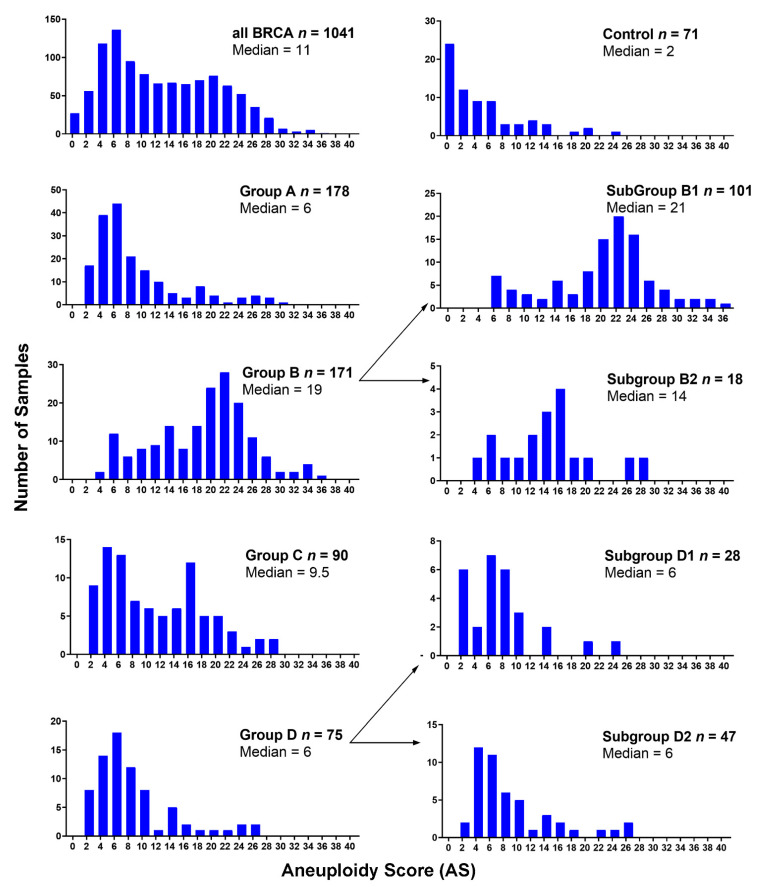
Distribution of the aneuploidy score (AS) for all BRCA samples and for each 1,16-chromogroup. Number of samples (*y*-axis) for different AS values (*x*-axis; bin equal to two) are shown. The total number of samples (*n*) and the median AS in each group is reported in the corresponding graph.

Figure 4 shows the distribution of the aneuploidy score (AS) (i.e., the number of arm-level aberrations per cancer sample) for each 1,16-chromogroup. The control group showed the lowest averaged AS (mean ± SD: 4.46 ± 5.53; median: 2; interquartile range (IQR): 0–6). It is interesting that an enrichment of samples with a low AS was observed in Group A (mean ± SD: 8.24 ± 6.32; median: 6; IQR: 4–10) and Group D (mean ± SD: 7.88 ± 5.75; median: 6; IQR: 4–9), while an enrichment of samples with a high AS was observed in group B (mean ± SD: 18.30 ± 6.96; median: 19; IQR: 13–23) and subgroup B1 (mean ± SD: 19.49 ± 6.82; median: 21; IQR: 16.5–23.5). Indeed, the increased AS in groups B/B1 was due to the higher frequency of arm-level aberrations, mainly losses, in several different chromosomes (Figure 3). An intermediate value of AS was observed in subgroup B2 (mean ± SD: 13.88 ± 6.27; median: 14; IQR: 9.5–16.25) and group C (mean ± SD: 10.53 ± 7.04; median: 9.5; IQR: 4–16) (Figure 4).

### 3.2. Transcriptomics in 1,16-Chromogroups: Chromosomal Distribution of Overexpressed Transcripts and Gene Dosage Effect

**Figure 5:**
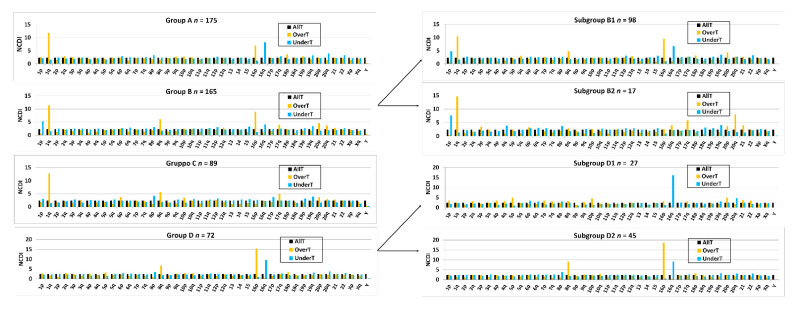
The normalized chromosomal distribution index (NCDI) of OverT (Overexpressed Transcript in comparison to CTRL) and UnderT (Underexpressed Transcript in comparison to CTRL of each 1,16-chromogroup. NCDI values of all transcripts (AllT) analyzed by RNA-seq in each chromosomal arm are also reported for comparison.

An analysis of differential expression of transcript levels between different 1,16-chromogroups was performed by the edgeR package [19,30] and expressed as the linear fold-change (FC) between a selected 1,16-chromogroup and the CTRL group (FCvsCTRL). The numbers of samples in each group are reported in Table 1 and Figure 5. We called OverT (Overexpressed Transcripts) or UnderT (Underexpressed Transcipts) those transcripts expressing a value of the FCvsCTRL >1.3 or <-1.3, respectively. OverT and UnderT were selected at a false discovery rate adjusted *p*-value (adjp) of <0.05. As shown in Figure 5, the chromosomal distribution of OverT and UnderT genes (expressed as normalized chromosomal distribution index (NCDI); [23]) was in agreement with the arm-level aberrations selected for each chromogroup (compare Figure 3 and Figure 5). In order to easily identify the modifications of the chromosomal distribution of OverT and UnderT, the chromosomal distribution of all transcript-encoding genes (*n* = 56,540) is also reported in Figure 5. For instance, Group A showed an increased density of OverT in 1q and 16p and a decreased density in 16q, in agreement with the fact that copy number aberrations in those chromosomal arms were used as criteria for the formation of such group (Figure 5). A similar correlation could be observed in all other 1,16-chromogroups. Such correlation extended to chromosomal arm aberrations that were not primarily selected during formation of the 1,16-chromogroups, such as the increased frequency of 8q gain in subgroups B1 and D2. This was an expected result that is easily explained by the well-known “gene dosage transcriptional cis-effect” reported in several published studies (see references in [22,23]).

In Table 2, we report the number of 1q-OverT (OverT encoded by genes located in chromosome 1q; FCvsCTRL > 1.3; adjp < 0.05) and 1q-UnderT (UnderT encoded by genes located in 1q; FCvsCTRL < −1.3; adjp < 0.05) in the examined 1,16-chromogroups. The OverT/UnderT ratio clearly distinguished groups bearing a combined 1q-gain and 16q-loss (A and B1) from those bearing only the 1q-gain (B2 and C) and those showing only a 16q-loss (D1 and D2). The number of 16q-OverT and 16q-UnderT are also reported in the same Table 2. As expected, an increased number of 16q-UnderT can be observed in groups bearing the 16q-loss (groups A, B1, D1, and D2).

In order to establish if the chromosomal rearrangements underlying arm-level aberrations were producing local effects on chromatin structure that are able to preferentially modify gene expression in specific chromosomal regions, we performed an analysis of the density of OverT and UnderT in specific cytogenetic bands of chr1 and chr16. As shown in Figure S2, the NCDI of OverT and UnderT in cytogenetic bands showed significant variations, but the pattern did not show any preferential telomeric or centromeric localization or gradient. Interestingly, 1q-gain bearing groups (A, B1/2, and C) showed a similar cytoband distribution pattern of OverT in chr1q, while they showed marked differences with groups not bearing 1q gain (D1/2). This observation was a consequence of the large number of shared 1q-OverT among groups A, B1, B2, and C (Table 2), thus confirming the similarity of the gene-dosage dependent transcriptional increase in those subgroups independently from the specific cytogenetic aberrations determining it. The chromosomal band distribution of UnderT in 16q was also consistent with the presence or absence of 16q-loss (note the relative enrichment of UnderT in band 16q13 in groups B2 and C bearing a 16q-disomy).

### 3.3. Hierarchical Clustering Based on Highly Significant OverT and UnderT Located on chr1 and chr16

**Figure 6:**
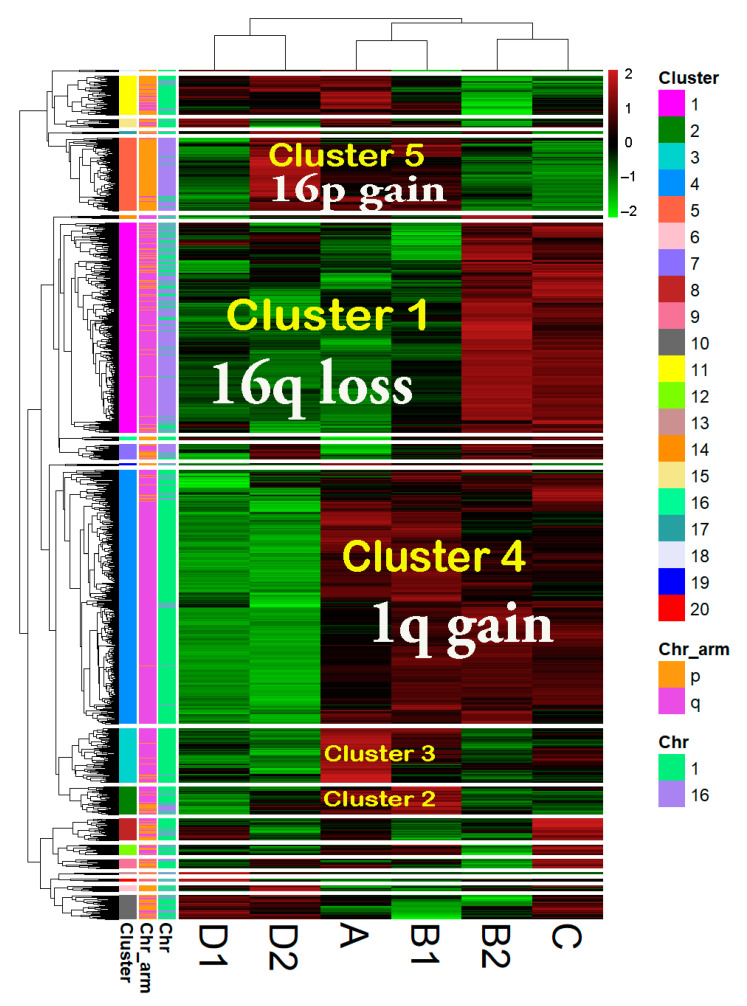
Hierarchical clustering of 1471 differentially expressed genes (DEGs) comparing group A vs. CTRL (FCvsCTRL > 1.3 or <−1.3 at adjp < 0.001) Only DEGs located on chromosome 1 and 16 were used for analysis. The transformation of “linear FC” to “modified linear FC” (modified linear FC is equal to “linear FC-1” if linear FC > 1 or to “linear FC + 1” if linear FC < 1) was performed in order to avoid the gap between −1 and +1 present in linear FC values. Data are clustered by the unweighted pair group method with arithmetic mean (UPGMA) with Euclidean distance. The common chromosomal abnormality among different groups is overwritten on the corresponding columns (in white letter). The cluster number is indicated by colors in the first column on the left and is repeated in some cases by overwriting the data columns (in yellow letters). Chr: chromosome.

We selected DEGs (FCvsCTRL > 1.3 or <−1.3 at adjp < 0.001) between group A and the CTRL group. We focused on DEGs located on chr1 and chr16, thus obtaining a list of 1471 DEGs that were denominated “1,16-A-DEGs” and that comprise 830 OverT (FCvsCTRL > 1.3) and 641 UnderT (FCvsCTRL < −1.3). In order to obtain a global comparison of the expression of 1,16-A-DEGs in 1,16-chromogroups, we performed a hierarchical clustering analysis using the “modified FCvsCTRL values” of the 1471 genes for each group (Figure 6; chromogroups in columns). A clear clustering of the groups according to similarity in the chromosome aberration pattern was observed. Group A and B1 clustered together in agreement with the fact that those groups harbor a concomitant 1q gain and 16q loss; group C and B2 clustered in accordance to the shared 1q-gain and disomic chr16, while the clustering of D1 and D2 reflected the shared 16q-loss and disomic chr1.

The clustering at the gene level (rows in Figure 6) clearly showed the formation of clusters corresponding to gene expression changes concordant with copy number changes. For instance, gene cluster 4 was found to contain genes located in chromosome 1q and overexpressed in 1q gain-bearing groups, gene cluster 1 was found to mainly be composed of underexpressed genes in 16q-loss samples, and gene cluster 5 was found to contain overexpressed genes in 16p-gain samples. Interestingly, clusters 3 and 2 were characterized by genes, mainly localized in chromosome 1q, that showed a higher expression in group A and B1, the two groups simultaneously bearing 1q-gain and 16q-loss. The values of the “modified FCvsCTRL” are reported in Figure S3 for some representative genes belonging to clusters 1, 3, and 4.

### 3.4. Integrated Cytogenomics and Transcriptomic Analysis: Selection of 1q-OverUpT and 16q-UnderT Genes

The global analysis in the previous paragraphs confirmed that the transcriptional gene-dosage effect is quantitatively relevant in establishing gene expression differences among the various 1,16-chromogroups, but it did not provide any hints on the molecular mechanisms involved in generating putative cancer driver effects. The sensitivity of different genes to the dosage effect may depend on the cellular context [31,32] and, in the case of cancer cells, on the specific cancer type or subtype. Moreover, it is likely that only a subgroup of those “dosage sensitive genes,” affected by recurrent cancer type-specific aneuploidies, are exerting driver effects. Several strategies have been previously devised to identify such genes [33,34,35,36,37,38]. In the present work, our strategy was founded on the hypothesis that functional interactions between the products of genes located in 1q and 16q can underlie the cancer driver effects of co-occurrent 1q-gain and 16q-loss. Such functional interactions can take place at several different levels, such as transcriptional regulation, non-coding RNAs interactions, protein interactions, post-translational modifications, post-translational degradations, and metabolic pathways. Transcriptome data from the different chromogroups and some arguments based on previous studies can provide a guide for the selection of putative driver genes located on 1q or 16q. The first argument is that some cancer driver genes are, indeed, located in chromosome arms 1q and 16q, and data supporting this statement were already reported in the introduction. The second argument is that transcriptional changes driven by gene-dosage are the only effects shown to be induced by der(1;16) or i(1q) up to now; other mechanisms, such as gene fusions, enhancer hijacking, or chromatin transcriptional dysregulation, have not been detected. The third argument is that cancer driver genes located on 1q/16q are likely to be differentially expressed, at the transcript level, between 1,16-chromogroup bearing 1q-gain and 16q-loss and the “CTRL group.”

Taking such arguments into account, what are the appropriate descriptors of the transcriptional dysregulation of 1q or 16q genes in the different 1,16-chromogroups? In the previous paragraph, we used the terms “overexpression” and “underexpression” to indicate increases or decreases of transcript levels comparing cancers bearing an arm-level aberration of chromosome 1 and 16 with a so-called CTRL group. The corresponding transcripts have been denominated as “OverT” and UnderT,” and the parameter used to describe quantitatively such differences has been called “fold-change vs. control” (FCvsCTRL). However, another important descriptor of cancer dysregulation relies on a comparison between the cancer group and the corresponding normal tissue. Increased or decreased transcripts in such comparison are indicated here as “Upregulated Transcripts” (UpT) or Downregulated Transcripts (DownT), and the corresponding quantitative parameter is the “fold-change vs. normal tissue” (FCvsN). In the following paragraphs, we use these parameters, alone or in combination, in order to select putative candidate driver genes associated with transcriptional increases linked to 1q-gain or to transcriptional decreases linked to 16q-loss.

As reported in Table 3, the three 1,16-chromogroups bearing a diploid 1q (D1, D2, and CTRL) showed an approximately equal number of 1q-located UpT and DownT (UpT/DownT ratio ranging from 1.0 to 1.6), while groups bearing the 1q-gain (A, B1, B2, and C) showed a higher number of UpT (UpT/DownT ratio ranging from 3.01 to 3.45). A relatively large number of genes (about 25–50% of cancer upregulated transcripts detected in 1q) were found to be specifically upregulated in 1q-gain bearing cancers. The number of UpT and DownT in chr16q are also reported in Table 3: as expected, the ratio UpT/DownT in 16q was lower in cancer groups bearing the 16q-loss (groups A, B1, D1, and D2).

**Figure 7:**
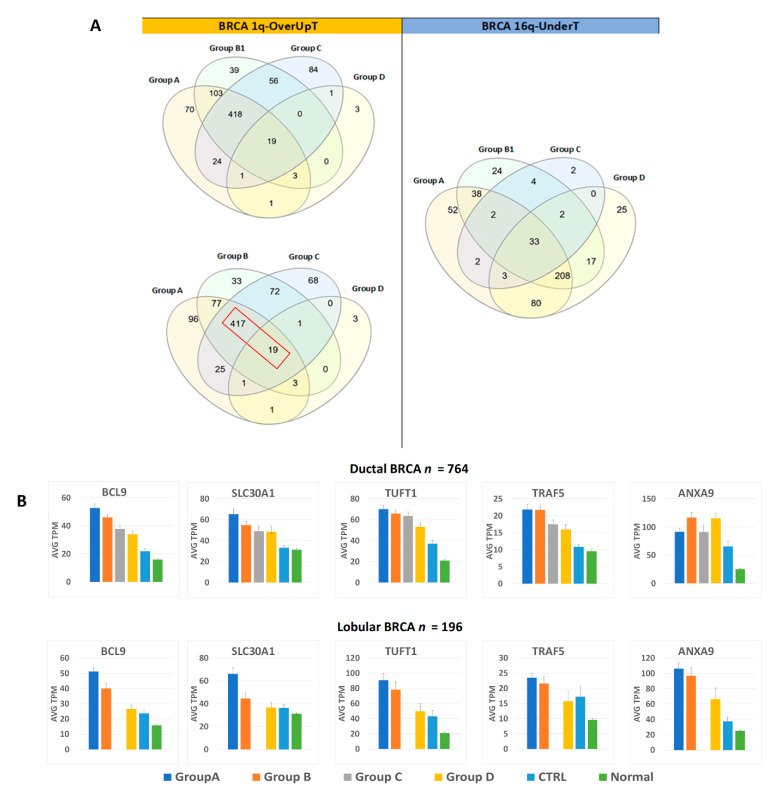
(**A**) Venn diagrams showing the number of shared 1q-OverUpT (transcripts that are overexpressed in comparison to CTRL and upregulated in comparison to normal tissue) (left panel) and 16q-UnderT (right panel) among different 1,16-chromogroups. The 1q-OverUpT shared among groups A, B, and C are indicated by a red box (436 genes). (**B**) Representative core 1q-OverUpT in ductal or lobular breast carcinomas; expression values are reported as averages (AVG) of TPM (transcripts per million) ± SEM. The total number of ductal and lobular cancer samples (*n*) is reported in the graph. Due to the rarity of lobular histotype in group C (one sample), no average value could be calculated and the corresponding column is absent in the graph.

Gene expression data were also analyzed by focusing on the comparison between the CTRL cancer group and each of the other 1,16-chromogroups, as described in the previous sections. Such comparison allowed for the classification of transcripts as OverT (overexpressed versus CTRL, FCvsCTRL > 1.3 at adjp < 0.05), a class enriched in gene-dosage-sensitive genes. However, OverT could be upregulated (OverUpT), downregulated (OverDownT), or not significantly changed in comparison to normal tissue. On the basis of previous studies [22,23], we only prioritized those genes that are overexpressed (FCvsCTRL > 1.3, adjp < 0.05) and upregulated (FCvsN > 1, adjp < 0.05) as candidate driver genes located in gained 1q and called them 1q-OverUpT. Out of 2410 transcripts located in chromosome 1q, 639 transcripts could be classified as OverUpT in group A (1q-A-OverUpT). Indeed, after identifying 1q-OverUpT in all chromogroups (see Table S2 for 1q-OverUpT in all 1,16-chromogroups), we observed that a large number of genes were shared among 1q-gain groups: 437 among groups A, B1, and C and 436 among groups A, B, and C (Figure 7A, left panel).

Moreover, the following nineteen 1q-OverUpT genes are also shared with the Group D (not bearing 1q-gain): TRIM46, SLC19A2, BCL9, CRABP2, SOX13, AL136987.1, SLC30A1, CIART, DENND1B, C1orf100, AL391001.1, TUFT1, TRAF5, PIAS3, AGT, IL19, CFAP45, F13B, and ANXA9. Those genes were called “core 1q-OverUpT,” and the expression values, reported as TPM, of representative transcripts are shown in Figure 7B.

In order to select candidate genes located in 16q that can cooperate with 1q-OverUpT, we prepared a list of UnderT genes in the comparison group A vs. control (FCvsCTRL < −1.3, adjp < 0.05). In the case of 16q-loss, the differential expression against the normal tissue was not included among the selection criteria because, as previously discussed by Condorelli et al. 2018 [22], a decreased expression of both upregulated and downregulated genes might play a significant role in cancer progression. Out of 1078 transcripts located in chromosome 16q, 418 transcripts could be classified as UnderT in group A (16q-A-UnderT). Indeed, after identifying 16q-UnderT in all 1,16-chromogroups (see Table S3 for 16q-OverUpT in all 1,16-chromogroups), we observed that a large number of genes (*n* = 208) were shared among 16q-loss groups (A, B1, and D) and other 80 genes among group A and D, as shown in the Venn diagram in Figure 7A (right panel).

### 3.5. Pathway Enrichment Analysis of 1q-OverUpT and 16q-UnderT Genes

**Figure 8:**
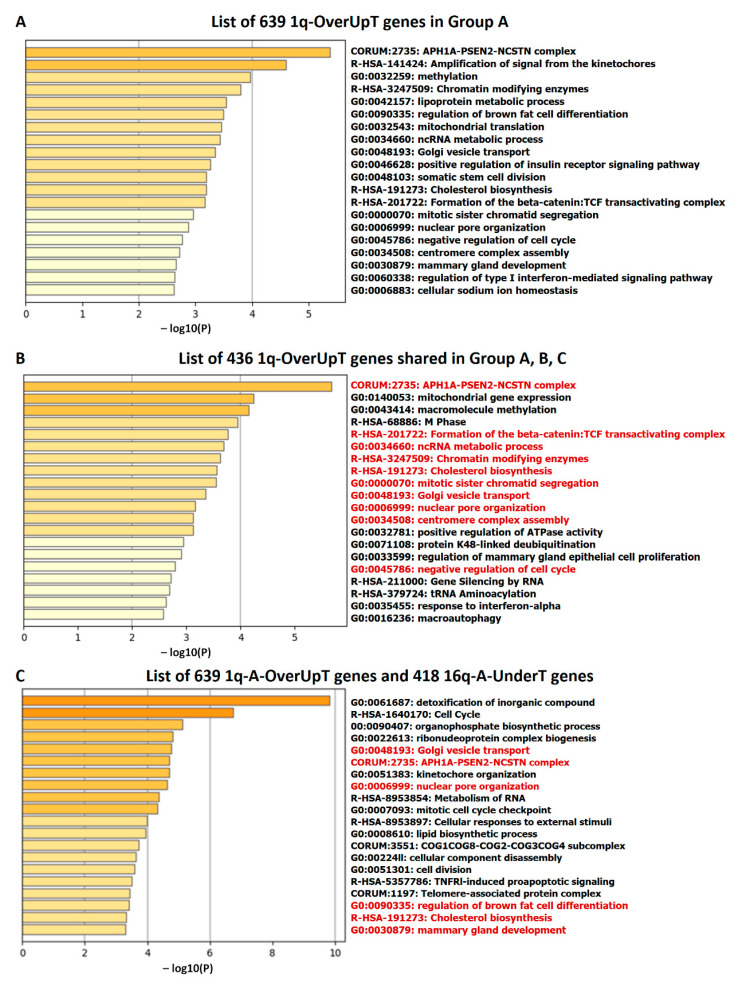
Pathway enrichment analysis using: (**A**) the list of 639 1q-OverUpT genes in group A, (**B**) the list of 436 1q-OverUpT genes shared among the three 1q-gain groups, and (**C**) the combined list of 639 1q-A-OverUpT genes (FCvsCTRL > 1.3 at adjp < 0.05 and FCvsN >1 at adjp < 0.05) and 418 16q-A-UnderT genes. Pathways in red letters in (**B**,**C**) are present also in (**A**).

In order to obtain information on functional pathways linked to 1q-OverUpT genes in group A, we submitted the list of 639 1q-A-OverUpT genes (FCvsCTRL > 1.3 at adjp < 0.05 and FCvsN > 1 at adjp < 0.05) to Metascape [26] and performed a pathway enrichment analysis and a protein–protein interaction analysis (PPI). Metascape identified all statistically enriched terms in the list using different knowledge-bases and the top 20 pathways are reported in Figure 8A. A similar pathway enrichment analysis was performed using the list of 436 1q-OverUpT genes (indicated by a red box in Figure 7A) shared by all 1q-gain groups A, B, and C. As shown in Figure 8B, the majority of top-ranking genes were shared among the two analysis, with the APH1–PSEN2–NCSTN complex as the top-first pathways in both lists.

In order to explore the functional cooperation among 1q and 16q genes, a combined list of 639 1q-A-OverUpT genes (FCvsCTRL > 1.3 at adjp < 0.05 and FCvsN >1 at adjp < 0.05) and 418 16q-A-UnderT genes was submitted to Metascape. The results of the pathway enrichment analysis are shown in Figure 8C (top 20 pathways). The complete list of significant terms hierarchically clustered into a tree based on Kappa-statistical similarities, and the terms within each cluster are reported in Table S4 (“Enrichment Analysis”). An analysis of pathways including 1q and 16q genes revealed a cooperation of the WWP2 gene (chr16q) in the “APH1A–PSEN2–NCSTN complex” (CORUM: 2735; one of the top-20 pathways in Figure 8) and “NOTCH3 Activation and Transmission of Signal to the Nucleus” (R-HSA-9013507) pathways.

**Figure 9:**
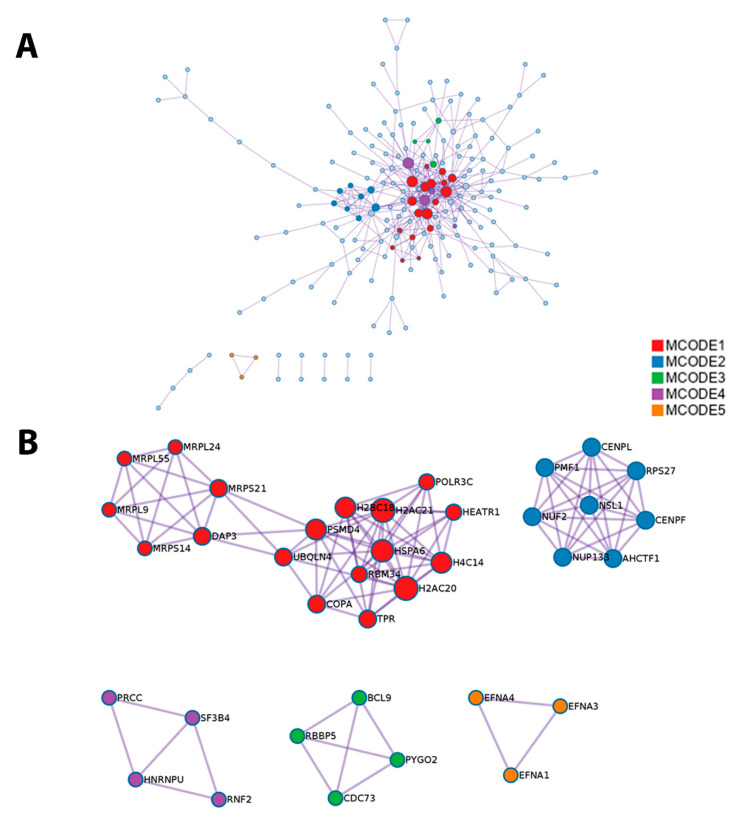
(**A**) Network of protein–protein interaction enrichment analysis in 1q-OverUpT genes in group A; (**B**) results of the application of the Molecular Complex Detection (MCODE) algorithm to this network. Networks were generated by Metascape.

The software Metascape also provided a protein–protein interaction enrichment analysis, which was carried out with the databases BioGrid, InWeb_IM, and OmniPathA, and generated a network using proteins that form physical interactions with at least another member in the list of 1q-OverUpT genes in group A (Figure 9A). The Molecular Complex Detection (MCODE) algorithm [39] was then applied to this network to identify regions where proteins are densely connected. GO (Gene Ontology) enrichment analysis was applied to each MCODE network to assign a functional meaning (Figure 9B).

### 3.6. Gene Set Enrichment Analysis (GSEA)

In a previous subsection, we determined whether the list of a special group of differentially expressed genes, called 1q-OverUpT and 16q-UnderT, was enriched for pathway or ontology terms. As is the case with over-representation methods, the results were dependent on the cutoff used in constructing the list. In this subsection, we report the results obtained with an additional tool for the analysis of genome-wide gene expression profiles, called GSEA [27]. GSEA takes the expression values of all transcripts, not only those above an arbitrary cutoff of fold-change or significance, into account. TPM values obtained in RNA seq analysis of 56,000 transcripts from 1058 samples were given as input to the GSEA software, and each cytogenetic group was compared with the CTRL group (Analysis I). Comparing group A vs. CTRL, 21 pathways from the REACTOME database showed an NES > 1.5 (Table 4), although none of those pathway showed an FDR (False Discovery Rate) value < 0.25. As indicated in Table 4, 20 out of 21 top-pathways included genes located in chromosome 1q among the leading edge subset.

Clues about functional interactions among transcriptionally dysregulated genes could be derived by the analysis of the pathways “NOTCH2 Activation and Transmission of Signal to the Nucleus,” “Deactivation of the beta-catenin transactivating complex,” and “Formation of the beta-catenin:TCF transactivating complex” (Table 4). Interestingly, the top-ranked genes in the two beta-catenin pathways were BCL9, PYGO2, RBBP5, and CDC73—the same genes forming one of the module identified by the MCODE algorithm in the PPI interaction network. The top-ranked genes in the “NOTCH2 Activation and Transmission of Signal to the Nucleus” were the genes APH1A, PSEN2, and NCSTN, whose products were subunits of the γ-secretase, a protease complex able to cleave various proteins within their transmembrane domains. Indeed, an increased expression of those genes in hormone receptor-positive breast cancers has been previously reported [40,41]. All these genes belong to the 1q-OverUpT group, and their pathways were already identified in the Metascape analysis reported in the previous subsection.

The top pathways (NES > 1.5) for each cytogenetic group are reported in Table S5. Among shared pathways between groups A and D or among groups A, B1, and D, several pathways, such as “Mitochondrial tRNA aminoacylation” and “Formation of Incision Complex in GG-NER,” include 16p- and 1q-located genes among the leading edge subset of GSEA analysis of both group A and D, and several genes, such as PARP1, IARS2, TARS2, and DARS2 belong to the 1q-OverUpT group. This result further confirmed that some 1q genes involved in cancer-activated pathways were also overexpressed in a cytogenetic group devoid of 1q-gain (group D) and might functionally cooperate with genes located in 16p. Therefore, such pathways may provide some clues about functional interactions induced by 1q-gain and 16p-gain aberrations. Indeed, 16p gain was found to be a frequent aberration shared by tumors of groups A, B1, and D. However, such GSEA analysis could not provide information about functional interactions between 1q and 16q genes because genes were ranked according to the real value of Signal2Noise (i.e., the difference of means of the two compared groups scaled by the standard deviation), a metric score that could take either positive or negative values. Genes modified by the transcriptional dosage-effect were expected to show positive scores if located in 1q-gain and negative scores if located in 16q-loss and to be ranked at the opposite ends of the list, thus preventing the identification of putative cooperative effect of 1q-OverT and 16q-UnderT genes. In order to overcome this issue, we repeated the analysis by sorting the genes using the absolute value of the Signal2Noise metric score. Many of the functional pathways associated with cytogenetic groups in the previous analysis (real value-analysis) were confirmed in the analysis based on the absolute value of Signal2Noise (absolute value-analysis, Table S6). In the group A analysis, “Formation of the beta-catenin:TCF transactivating complex,” “Deactivation of the beta-catenin transactivating complex,” “Mitochondrial tRNA aminoacylation,” “NOTCH2 Activation and Transmission of Signal to the Nucleus,” “NOTCH3 Activation and Transmission of Signal to the Nucleus,” and “Signaling by NOTCH2” showed an NES > 1.5. In this analysis, the pathway “Ephrin mediated repulsion of cells” ranked among the top positions due to the 1q-located genes EFNA1, EFNA4, and EFNA3 and the gamma secretase components APH1A, PSEN2, and NCSTN. Indeed, several pathways were shared among different cytogenetics groups (Table S6). The pathway “NOTCH3 Activation and Transmission of Signal to the Nucleus” was enriched in group A (NES = 1.71, nominal *p*-value = 0.001, FDR *q*-value = 0.28), B1 (NES = 1.75, nominal *p*-value < 0.001, FDR *q*-value = 0.11), group B2 (NES = 1.52, nominal *p*-value = 0.005, FDR *q*-value = 0.30), group C (NES = 1.75, nominal *p*-value < 0.001, FDR *q*-value = 0.04), and group D2 (NES = 1.61, nominal *p*-value < 0.001, FDR *q*-value = 0.12).

The absolute score-analysis allowed for the identification of pathways including 1q and 16q-located genes among the leading-edge subsets. After excluding the pathways showing a discordant functional effect of 1q-OverT genes and 16q-UnderT genes, such analysis suggested possible cooperative functional interactions in the pathway “NOTCH3 Activation and Transmission of Signal to the Nucleus” in groups A, B1, and D2. In the “NOTCH3 Activation and Transmission of Signal to the Nucleus” pathway, the overexpression of three 1q genes encoding subunits of the gamma secretase complex (APH1A, PSEN2, and NCSTN), which plays a positive role in NOTCH3 signaling, was found to be functionally interconnected with the reduced expression of the 16q gene WWP2, an E3 ubiquitin ligase that negatively regulates NOTCH3 signaling [42]. Another pathway implicating a functional cooperation between 1q genes and 16q genes was the “Nuclear Signaling by ERBB4” enriched in group D2 (NES = 1.61, nominal *p*-value: <0.001, FDR *q*-value = 0.11). The involved 1q gene was APH1A and the 16q gene was WWOX, a WW-domain-containing protein that binds to a cytosolic fragment of ERBB4 (generated by the gamma secretase complex) and prevents its translocation to the nucleus [43].

### 3.7. Analysis II. Subclassification of 1,16-Chromogroups in Histological and Molecular Subtypes and Differential Gene Expression among 1,16 Chromogroups Containing Only Ductal Adenocarcinomas of the LumA Subtype

**Figure 10:**
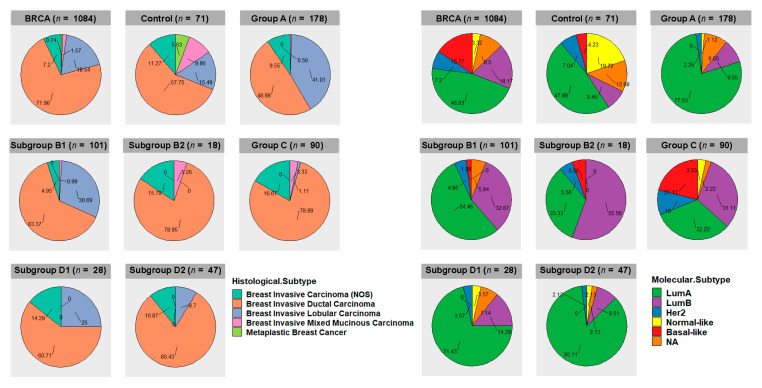
Frequencies of histological subtypes (**left** panels) and molecular subtypes (**right** panels) in the different 1,16-chromogroups.

In this work, we compared tumors bearing a specific arm-level aberration (study group) to tumors not bearing it (control group). The basic assumption was that part of the gene expression differences between the two groups were linked to the presence of the chromosomal aberration. However, the choice of criteria used for the generation of the control cancer group exerted a strong impact on the results. Indeed, two important points should be taken into account: (1) although tumors were found to belong to the same clinico-pathological type (breast invasive carcinoma), phenotypic and genotypic heterogeneity was present in all cytogenetic groups; (2) cancers in the so-called “control group” differed from those in the study group, not only for the absence of the specific chromosomal aberration but also for other mutational events and carcinogenesis pathways. Regarding the first point, it is possible to classify breast invasive carcinomas in histological subtypes using classical optical microscopy or in molecular subtypes using specific molecular biomarkers and transcriptome analysis [44,45]. The frequencies of histological subtypes and molecular subtypes in the different cytogenetic groups are reported in Figure 10. Invasive ductal carcinoma was found to be the predominant histological subtype in all cytogenetic groups. Invasive lobular carcinomas showed the highest frequency in group A and were associated with cytogenetic groups bearing 16q-loss (A, B1, D1, and D2). Molecular subtype LumA was enriched in cytogenetic groups A, B1, D1, and D2, while LumB was enriched in subgroup B2. The basal-like subtype showed the highest frequency in the cytogenetic group C, while the normal-like subtype showed the highest frequency in the control group.

**Figure 11:**
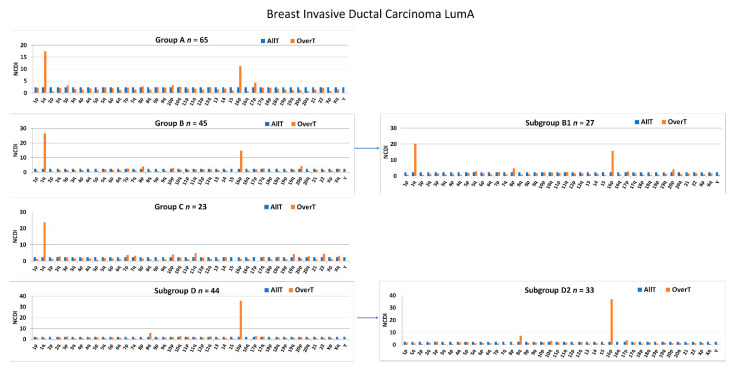
NCDI values of OverT of each 1,16-chromogroup formed with breast invasive ductal carcinomas of the LumA subtype. NCDI values of all transcripts (AllT) analyzed by RNA-seq in each chromosomal arm are also reported for comparison.

Therefore, in order to reduce phenotypic and genotypic heterogeneity, we repeated all the transcriptome analyses by selecting only the most common histological and molecular subtypes (breast ductal carcinoma and LumA, respectively). The available number of breast ductal LumA carcinomas allowed for the generation of group A, B, B1, C, D, and D2 (Figure 11). As expected, the NCDI values of 1q and 16p were increased in samples bearing 1q-gain and 16p-gain. The increased NCDI value of 8q was still detectable in subgroup D2.

The GSEA analysis was repeated using transcriptome data derived from 310 breast ductal LumA carcinomas (herein called “Analysis II” in order to distinguish it from “Analysis I” reported in the previous subsection; see Table S1 for sample details). In Analysis II, the comparison of group A vs. CTRL identified 23 REACTOME pathways showing an NES > 1.48 (Table 5).

Analyses I and II showed a good overlapping of the 50 top-NES-ranked pathways in all groups (50% group A; 38% group B1, 42% group D2), except for group C (8%). The inclusion of a relatively large group of basal-like cancers in group C in “Analysis I” was the likely explanation for this discrepancy.

**Figure 12:**
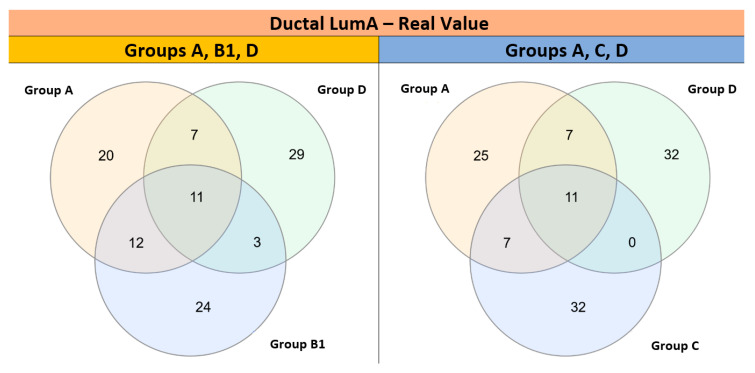
Venn diagrams showing overlapping of top-ranked functional pathways identified in GSEA Analysis II (restricted to 310 invasive ductal carcinomas of the LumA subtype) among different chromogroups.

Interestingly, “Analysis II” showed a large number of shared pathways (*n* = 11) among groups A, B1, and D or A, C, and D (Figure 12). Indeed, Analysis II (restricted to Ductal LumA cancers) confirmed several pathways previously identified in “Analysis I,” such as “Mitochondrial tRNA aminoacylation,” “Deactivation of the beta-catenin transactivating complex,” “Formation of the beta-catenin:TCF transactivating complex.” Moreover, the enrichment of “Formation of the beta-catenin:TCF transactivating complex” (NES = 1.52, nominal *p*-value = 0.007; FDR *q*-value = 0.206) and “EPH-ephrin mediated repulsion of cells” (NES = 1.52, nominal *p*-value < 0.001; FDR *q*-value = 0.211) were statistically significant in Analysis II performed with absolute Signal2Noise values (Table S6).

### 3.8. Analysis III: Cytogenomics and Transcriptomics Differences of Ductal and Lobular Breast Cancer Classified in 1,16-Chromogroups

A detailed molecular characterization of the differences between invasive ductal and lobular carcinomas had been previously provided by integrated omics analysis [45,46]. The higher frequency of CDH1 loss-of-function mutations and the lower transcriptional expression of the CHD1 gene in lobular carcinomas were some of the main differences between the two histotypes. Indeed, a decreased expression of CDH1, at both the protein and transcript levels, was probably underlying the discohesive phenotype of lobular carcinomas.

**Figure 13:**
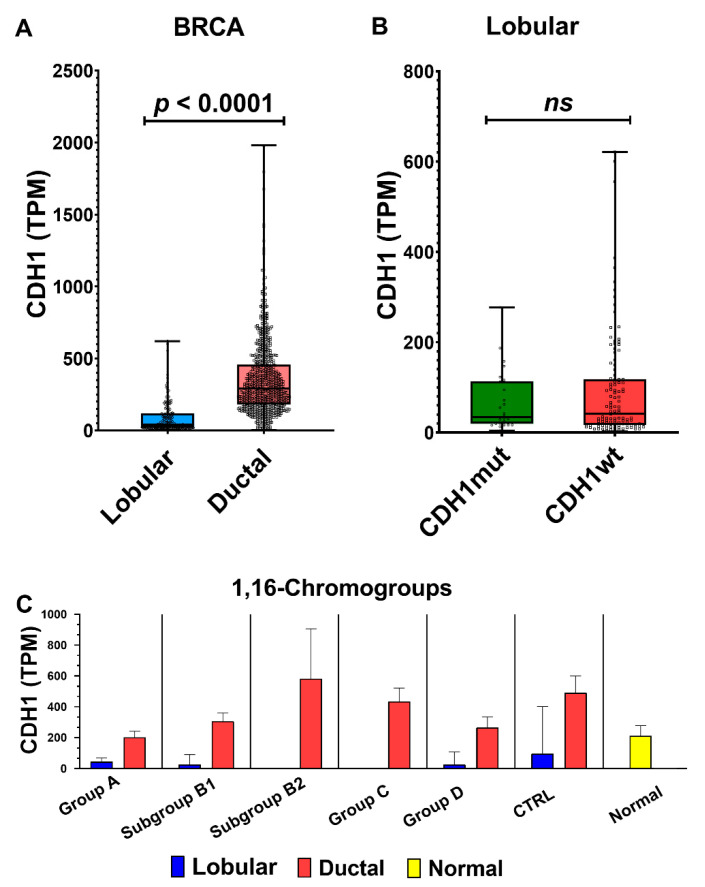
(**A**) Dot plots of CDH1 transcript levels in lobular and ductal carcinomas and (**B**) in lobular cancer bearing a mutated CDH1 (CDH1mut) or a wild-type CDH1 (CDH1wt); overlaid boxes show median and interquartile range; statistical significance by Mann–Whitney test; (**C**) TPM levels of CDH1 in different 1,16-chromogroups differentiated in lobular and ductal cancers. Columns represent the median values, and bars represent the 95% confidence intervals. ns: not statistically significant.

**Figure 14:**
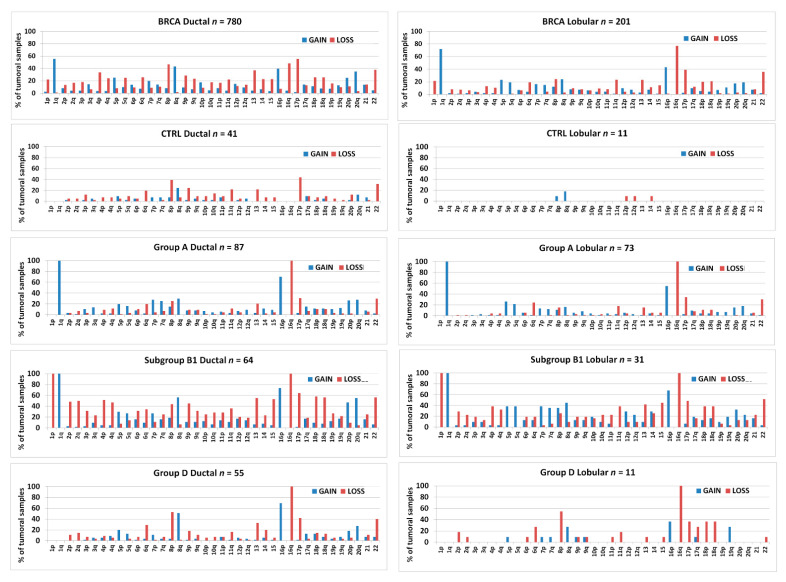
Bar graphs showing the percentage of samples bearing arm-level gains or losses in chromosomes 1–22 in invasive ductal or lobular breast carcinomas or in the different 1,16-chromogroups formed with invasive ductal carcinomas or invasive lobular carcinomas, as indicated in each graphs. The number of samples (*n*) in each chromogroup is reported in the corresponding graph.

We confirmed the clear-cut difference in CDH1 expression between ductal and lobular carcinomas (Figure 13A). In this work, we could also compare the transcript levels of CDH1 in breast cancer groups differentiated by the presence or absence of Chr1 and 16 aberrations (Figure 13C). As already shown in Figure 10, the 1,16-chromogroups characterized by 1q-gain and 16q-disomy (groups B2 and C) had no or few invasive lobular carcinomas, thus precluding the analysis of such a cancer histotype in those two groups. However, the CTRL group of ductal carcinomas (i.e., without abnormalities of Chr 1 and 16) showed a sufficient number of both histotypes (40 ductal vs. 11 lobular carcinomas), thus allowing for comparisons with all other breast cancer groups and with corresponding normal tissue. Interestingly, invasive ductal carcinomas with 16q-disomy (CTRL, B2, and C) showed a higher level of CDH1 transcripts in comparison to normal breast tissues, while CDH1 levels in invasive ductal carcinomas with 16q-loss (group A, B1, and D) were similar to those of normal tissue. This observation was in agreement with previous immunohistochemistry studies showing that low-grade invasive ductal carcinomas have stronger E-cadherin membrane staining than that seen in the normal breast epithelial cells, while E-cadherin loss may occur as a late event in a subgroup of high-grade invasive ductal carcinomas [47,48]. On the contrary, E-cadherin loss is observed as an early event in lobular carcinomas [49]. In the present work, we found that CDH1 transcript levels were lower in invasive lobular carcinomas either in 16q-loss groups (A, B1, and D) and in the 16q-disomic CTRL-group when compared to corresponding ductal carcinomas or to normal breast tissue (Figure 13C). In other words, invasive lobular carcinomas had a CDH1 expression lower than ductal carcinomas both in the presence (groups A, B1, and D) and the absence of 16q-loss (CTRL group). This observation suggested that additional mechanisms, besides the 16q-loss, are downregulating CDH1 expression in lobular carcinomas. Though it was clear that loss-of-function point mutations of CDH1 are cooperating to the decreased functionality of E-cadherin in lobular carcinomas, the transcript levels of lobular CDH1-mutated cancers were not significantly different from those of CDH1-wild type ones (Figure 13B). Indeed, it has been repeatedly suggested that other mechanisms, such as epigenetic modifications, the upregulation of CDH1 transcriptional repressors, and other forms of transcriptional dysregulation, may account for the downregulation of CDH1 transcription in lobular carcinomas [45,48]. Our analysis confirmed that the transcriptional downregulation of CDH1 in ductal carcinomas is weaker than that in lobular ones. Though 16q-loss is frequently observed both in ductal and lobular carcinomas (with a slight higher frequency in lobular ones; see Figure 14), the CDH1 transcriptional difference between the two histotypes was detectable both in the presence (group A, B1, and D) or the absence of 16q-loss aberrations (CTRL group; Figure 13C). Indeed, in the CTRL breast cancer group, it was possible to observe a relevant number of invasive lobular carcinomas in the absence of 16-q loss, and it is interesting to note that this 16q-disomic lobular subtype was found to be characterized by a near-euploid karyotype (Figure 14). Nevertheless, 16q-loss is a strong determining factor for the generation of invasive breast lobular carcinomas, as suggested by the lack or the rarity of this histotype in groups B2 and C, bearing 1q-gain but not 16q-loss (Figure 10). In conclusion, our analysis suggested that 16-q loss can be considered a critical chromosomal abnormality for the generation of lobular carcinomas in the context of a significant aneuploidy score (>4) (see Figure 14, which shows a comparison of arm-level chromosomal aberrations in lobular carcinomas of the CTRL group with those in lobular carcinomas of group A, B1 and D).

Moreover, invasive lobular carcinomas were found to be able to bear 16q-loss either with (group A and B1) or without 1q-gain (group D), in agreement with previous reports [45,46]. Indeed, the increased frequency of 16q-loss in lobular carcinomas was found to be accompanied by an increased frequency of 1q-gain, confirming that the co-occurrence of 1q-gain and 16q-loss is a feature of both ductal and lobular carcinomas in a larger sample population (Figure 14; also note the increased frequency of 8p-loss and 8q-gain in ductal carcinomas).

Statistically significant DEGs between lobular and ductal carcinomas (LvsD-DEGs) were also analyzed by the EdgeR software separately for groups A, B1, and D (decreased in lobular carcinomas: linear fold change lobular vs. ductal <−1.5 and adjp < 0.05; increased in lobular carcinomas: linear fold change lobular vs. ductal > 1.5 and adjp < 0.05). 16q-DEGs shared across all 16q-loss groups (A, B1, and D) are reported in Table S7. As expected (see also Figure 13C), CDH1 was significantly decreased in lobular carcinomas vs. ductal carcinomas in all 16q-loss groups (A, B1, and D) and was the only 16q-LvsD-DEGs that was coherently decreased in lobular carcinomas of those three groups. Moreover, CDH1 was the only gene belonging to the class of 16q-UnderT that was further decreased in lobular carcinomas in 16q-loss groups (Table S7). 16q-LvsD-DEGs were found to represent only a minor fraction of 16q-UnderT: only 3 transcripts out of 208 were LvsD-DEGs (AC040162.3, CDH1, IL34) UnderT shared across groups A, B1, and D (Table S7). In summary the analysis of LvsD-DEGs revealed very few specific transcriptional dysregulations superimposed to the common 16q-loss dependent downregulation, besides the known CDH1 downregulation.

A similar analysis was performed for 1q-LvsD-DEGs by selecting those genes showing concordant changes across 1q-gain groups (A and B1; Table S7). Again, those 1q-LvsD-DEGs represented only a minor fraction of OverUpT genes shared among groups A and B1 (10 1q-LvsD-DEGs out of 540 OverUpT shared between groups A and B1). Moreover, none of the “core 1q-OverUpT” genes showed a differential expression between ductal and lobular carcinomas, suggesting that their putative functional role in carcinogenesis might be shared between the two histotypes.

Given that the largest number of lobular carcinomas was observed in group A (bearing both 1q-gain and 16q-loss), it is reasonable to hypothesize that cooperative functional networks of 1q and 16q genes could operate both in lobular and ductal carcinomas, the two histotypes being mainly differentiated by the deeper transcriptional downregulation of 16q-CDH1 in lobular carcinomas.

### 3.9. Recurrent Point Mutations in Breast Cancer 1,16-Chromogroups

Recurrent point mutations by WES data are shown in oncoplots of Figures S4 and S5. The main histological subtypes (ductal or lobular carcinoma) are indicated by the annotation bar below the graph. The higher frequency of TP53 mutations in ductal carcinomas and CDH1 mutations (mainly nonsense or splice-site mutations) in lobular carcinomas was found to be a general feature of those histotypes, as shown in the oncoplot of Figure S4, including 709 ductal and 149 lobular BRCA samples analyzed by WES in TCGA study. An accurate analysis of recurrent point mutations in invasive ductal and lobular carcinomas has been already provided by previous studies using both TCGA and other data, and it is not repeated here [45,46,50]. However, the oncoplots in Figure S5 provide a rapid overview of the top 30 recurrent point mutations detected by WES in the different 1,16-chromogroups examined in the present study. It is clear that this type of presentation did not allow for a direct comparison between ductal and lobular cancers because of the largely different number of samples of the two histotypes in the different chromogroups (note the absence of lobular cancers in Group B2 and C, as already shown Figure 10). Nevertheless, a higher frequency of TP53 (nonsense, missense, and splice site) and GATA3 mutations (nonsense and splice site) in ductal carcinomas and CDH1 mutations in lobular carcinomas was easily recognizable in cytogenetic group A. Mutations of CBFB (mainly missense mutations) were most frequently detected in ductal carcinomas of group A and B1, while mutations of MAP3K1 (nonsense and missense) and ARID1A (missense and splice site mutations) were most frequently detected in ductal carcinomas of group D. PI3K mutations (missense) were found to be the most frequent mutation in all the chromogroups, with the exception of group C, where TP53 mutations (nonsense, splice sites, and missense) predominated in agreement with the higher level of chromosomal aberrations detected in this group. All those mutations were reported as significant in ductal or lobular cancers, or both, in previous analysis of TCGA data [45].

## 4. Discussion

Previous studies [9,10,11,51] have described the biological features of breast cancers showing simultaneous chromosome 1q-gain/16q-loss, reporting an association with steroid receptor presence and low proliferation in breast carcinoma. In an integrated view of genome and transcriptome from a large number of breast tumors, Curtis et al. (2012) [12] reported the identification of novel biological subgroups by the joint clustering of copy number and gene expression data in a discovery set of 997 breast cancers. In this analysis, an unsupervised clustering methodology suggested 10 different subgroups: one of these subgroups was characterized by the classical 1q-gain/16q-loss (called IntClust8; *n* = 143 samples), and another subgroup was characterized by the 16p-gain/16q-loss in the absence of 1q alterations (IntClust7, *n* = 109). However, no attempt to distinguish the different cytogenetic chromosome alterations that could generate the final chromosome 1q and 16q imbalances was made in those studies.

Previous large-scale studies aimed to obtain a molecular stratification of breast cancer useful for clinical management and for a global view on general mechanisms of breast cancer development and evolution [10,12,51,52,53]. In our work, we focused on a specific subpopulation of breast cancers (with 1q-gain and/or16q-loss), and our cytogenomics classification was simply an investigational tool for mechanisms involving transcriptionally dysregulated 1q and 16q genes. We exploited the availability of molecular cytogenetics data obtained using SNP-array technology [54,55] from a large number of TCGA samples in order to classify BRCA adenocarcinomas in different “chromogroups” according to the presence of different combinations of Chr 1 and 16 copy number abnormalities. Those combinations were designed to correspond to the expected patterns of copy number abnormalities generated by chromosomal aberrations previously found in classical cytogenetic studies of breast cancer, such as der(1;16) (q10;p10), i(1q), and del(16q). Indeed, the association of 1q-gain and 16q-loss can be produced by the single chromosomal aberration, such as der(1;16), or by the combination of two different aberrations, such as i(1q) and del(16q). The analysis of aneuploidy scores revealed an enrichment of samples with low values (median 6) in group A, whose copy number criteria were inspired by der(1;16), or in subgroup D1, inspired by del(16q), in agreement with the observation that those aberrations are often observed as the sole cytogenetic anomalies in breast cancer [1,2,3,4,5,6,7,8,9,11]. Higher aneuploidy scores were observed in samples with 1q-gain without aberrations of chr16 (groups C or B2: median scores of 9.5 and 14, respectively) or with concomitant 1q-gain and 16q-loss determined by two different co-occurring cytogenetic abnormalities such as i(1q) and del(16q) (subgroup B1: median score of 21). The latter observation suggested that a more complex evolutionary process, based on chromosomal instability, is involved in the progression of cancers of subgroup B1. Nonetheless, the relatively high frequency of tumors potentially bearing the der(1;16) (group A included 36% of all tumors showing 1q-gain or 16q-loss) and the fact that those tumors bear a low number of other chromosomal aberrations (low aneuploidy score) supported the hypothesis that genes located in 1q and 16q might play a strong cooperative cancer driver effect. In early cytogenetic studies in breast cancer, the higher pathogenic impact of 1q-gain was inferred from the observation that the two most frequent chromosomal aberrations, der(1;16) and i(1q), have 1q in common [1,2,56] However, following studies supported the importance of 16q-loss in the absence of chr1 aberrations in breast cancer [9,57,58] The data analysis performed in the present study confirmed the relatively high frequency of 16q-loss without chr1 aberrations and with a low aneuploidy score (group D included 15% of all tumors showing 1q-gain or 16q-loss).

Among the genes located on chromosome 16q, the CDH1 gene, encoding for the cell adhesion glycoprotein E-cadherin, has been repeatedly implicated as an important player in mediating the effect of 16q-loss in breast cancer. Indeed, CDH1-inactivating mutations have been found in 15–56% of invasive lobular breast carcinomas, and the majority of such mutations are associated with 16q-loss, thus generating the typical biallelic inactivation of tumor suppressor genes [45,46,59,60,61]. On the contrary, invasive ductal breast carcinomas rarely harbor CDH1-inactivating mutations [59,60]. However, both histological subtypes have shown a frequent 16q-loss, independently by the presence of inactivating point mutations of CDH1, and the invasive lobular carcinomas have shown a reduced expression of CDH1 at both the mRNA and protein level [45,48,59,62,63]. Indeed, other mechanisms for the reduced expression or function of E-cadherin, such as transcriptional downregulation, promoter methylation of CDH1, and post-translational modifications of E-cadherin, have been detected in breast cancer [61,64,65,66,67,68]. It is well-known that E-cadherin antigen, detected by immunohistochemistry analysis, is mainly expressed in ductal carcinomas and absent in lobular ones [66,69,70,71]. In agreement with immunohistochemistry results, a previous analysis of TCGA data [45] reported that CDH1 transcript and protein levels are significantly lower in lobular carcinomas compared to ductal ones, confirming that CDH1 expression differentiates the two histological subtypes. However, Ciriello et al. (2015) [45] did not detect significant DNA hyper-methylation at the CDH1 promoter, excluding a role for such epigenetic modification in the observed CDH1 downregulation. In the present work, we reported that the CDH1 differential expression between ductal and lobular carcinomas was maintained in all examined 1,16 chromogroups that included a significant number of both histotypes (Figure 13). Collectively, these data indicated that transcriptional decrease of CDH1 is only a weak effect in invasive ductal carcinomas, suggesting that the frequent loss of 16q in such histological subtype might be explained by the cancer evolutionary advantage due to reduced transcription of other 16q genes [72] or by a higher sensitivity to CDH1 haploinsufficiency due to the cooperative effect of other gene mutations.

In light of the frequent association of 1q-gain and 16q-loss in both invasive and lobular breast carcinomas, we reasoned that investigation of cooperative functional links between transcripts encoded in those chromosomal arms was a valid strategy for the identification of novel candidate driver genes underlying the selection of those recurrent chromosome aberrations. Previous studies, aimed to identify driver genes in 1q and 16q, separately analyzed expression of genes located in these chromosomes [3,72]. Indeed, the chromogroup A, enriched in der(1;16), was characterized by a low aneuploidy score, thus suggesting that the putative interchromosomal 1q/16q gene cooperation can be an early event and is not associated with an extensive chromosomal instability. Such cooperation might involve the early E-cadherin loss in lobular carcinomas [49]. On the contrary, E-cadherin loss occurs as a late event in invasive ductal carcinomas [47,48], and the understanding of the functional meaning of the early and frequent 1q-gain/16q-loss co-occurrence requires the definition of further mechanisms.

In order to select putative cancer driver genes located in chr1q and chr16q, we exploited comparisons of the corresponding transcript levels between chromogroups bearing 1q-gain and/or 16q-loss and a so-called “CTRL group,” i.e., a cancer group devoid of any arm-level aberrations of chr1 and chr16. The main assumption for this strategy was that the analysis of differential expression between those cancer groups could identify transcriptionally dysregulated genes sensitive to gene dosage effect and that this subset of genes is enriched in cancer driver genes.

With this in mind, we investigated functional cooperation between genes located in 1q and 16q by two different methods. In the first one, the selection of candidate genes was based on pre-established thresholds (linear FC and adjp values) in the comparisons between the selected 1,16-chromogroup and the CTRL group or between the selected chromogroup and the normal breast tissue group. Moreover, the concordance of transcript level changes with corresponding copy-number abnormalities in the different 1,16-chromogroups was taken into account. Based on previous data on aneuploidy-induced transcriptional changes [22,23], we selected OverUpT genes located in 1q and UnderT genes located in 16q and submitted those gene lists to the over-representation analysis of functional pathways [26]. In the second strategy, differential gene expression between different chromogroups and the “CTRL cancer group” was investigated by gene set enrichment analysis [27]. Both methods showed concordant results, pointing out the involvement of functional pathways that show the cooperation of genes located on 1q and 16q, such as “NOTCH2 Activation and Transmission of Signal to the Nucleus,” “NOTCH3 Activation and Transmission of Signal to the Nucleus,” and “Formation of the beta-catenin:TCF transactivating complex.” Indeed, the involvement of Notch signaling system in breast cancer has been repeatedly suggested in the literature (for a review see Mollen et al. 2018 [73]).

NOTCH signaling is a cell-to-cell communication system composed by transmembrane Notch receptors (Notch1–4) and transmembrane ligands (Delta/Jagged). After ligand binding, Notch receptors undergo conformational changes that expose a proteolytic site in the extracellular region. After cleavage, the remaining membrane fragment is cleaved at an intramembrane (inner leaflet) site by the gamma secretase complex, thus generating a soluble Notch-intracellular domain (NICD) that is able to translocate to the nucleus activating a specific transcriptional program. Some subunits of the gamma-secretase complex were found to be encoded by genes located on 1q (APH1A, PSEN2, and NCSTN) and were overexpressed and upregulated in cytogenetic groups A, B, and C (1q-gain bearing groups). Indeed, it has been previously shown that NCSTN (nicastrin) is overexpressed in breast cancer, and its genetic depletion is sufficient to inhibit tumor growth in vitro and in vivo [41]. The increased NCSTN copy-number, due to 1q-gain, can enhance other transcriptional and post-transcriptional mechanisms, thus leading to hyper-activation of gamma-secretase and NOTCH signaling in breast cancer [74]. The results of pathway analysis revealed an interesting functional link between gamma secretase genes, located on 1q, and genes located on 16q. WWP2 is a 16q gene that encodes an E3 ubiquitin-protein ligase acting on Notch3-NICD and targeting it to an endosomal/lysosomal degradation fate [42]. Indeed, the decreased WWP2 expression, associated with 16q-loss, can contribute to the pathological hyperactivation of Notch3-dependent gene expression. A role of Notch3 hyperactivation has been also shown in experimental models of breast ductal cancerogenesis [75,76,77].

Another functional relationship between 1q and 16q genes was provided by the demonstration that E-cadherin itself is one of the substrates of gamma-secretase [78]. Gamma-secretase cleavage dissociates E-cadherin from the cytoskeleton, thus promoting the disassembly of the adhesion complex and increasing the cytosolic pool of beta-catenin. Therefore, the cleavage of E-cadherin by gamma-secretase subunits encoded in Chr1q and the decreased transcription of the CDH1 gene produced by 16q-loss can represent cooperating mechanisms underlying the decreased function of adherens junctions in breast cancer. Moreover, the increase of cytosolic beta-catenin, due to adhesion complex disassembly, allows for its translocation to the nucleus where beta-catenin plays a crucial role in the so-called “Wnt enhanceosome.” Such a multiprotein complex, containing beta-catenin, BCL9, Pygo, and TCFs (T cell factors), activates the transcriptional program of Wnt signaling [79]. BCL9 functions as a scaffold of the Wnt enhanceosome by binding to the Pygo protein and to the N-terminus of the armadillo repeat domain of β-catenin, as well as by stabilizing the interactions of beta-catenin with TCF bound to cis-regulatory enhancers of Wnt-responsive genes [80].

Elsarraj et al. (2015) [81] showed a role for BCL9 in the transition from in situ to invasive ductal breast carcinoma and reported that BCL9 knockdown is able to inhibit the proliferation, migration, and invasion of ductal carcinoma in situ cells in vitro and in vivo breast cancer models. Moreover, they also analyzed TCGA gene expression data reporting that the Wnt/β-catenin pathway is significantly upregulated in BCL9-high cancers compared to BCL9-low breast cancers. In agreement with those data, our present analysis of OverUpT 1q genes and UnderT 16q genes pointed out the possible functional relevance of the pathway “Formation of the beta-catenin:TCF transactivating complex.” In particular, both BCL9 and its interacting partner, PYGO2, are located on chr1q and are overexpressed and upregulated in 1q-gain cytogenetic groups. Interestingly, BCL9 belongs to a small subset of 1q genes that were also found to be overexpressed and upregulated in group D, a cancer group bearing 16q-loss and 1q-disomy. Indeed, other mechanism linking BCL9 to Wnt signaling have been described, such as the ability to inhibit clathrin-mediated degradation of LRP6 signalosome components [82].

The hypothesis of a central role of BCL9 and Wnt signaling pathway may have distinct functional implications in the pathogenesis of ductal and lobular breast cancers. As previously discussed, the profound downregulation of E-cadherin is considered a hallmark of lobular cancer cells, and it has been reported that the loss E-cadherin in lobular cancers is associated with the destabilization of the beta-catenin protein, resulting in impaired canonical Wnt signaling [67,83,84]. On the other hand, a decreased functionality of E-cadherin can be achieved in ductal carcinomas by an excessive gamma-secretase processing (as hypothesized above), but this kind of mechanism can partially preserve E-cadherin membrane expression and function and its consequences on beta-catenin nuclear translocation have not fully investigated. Several research papers have reported an increased nuclear beta-catenin accumulation and an increased activity of beta-catenin dependent transcriptional activity in breast cancer [85,86,87,88,89,90,91,92]. However, the nuclear accumulation of beta-catenin or increased beta-catenin dependent transcription have only been detected in subgroups of breast cancers identified as triple-negative ones. Since our analysis revealed an overexpression of BCL9 in estrogen-receptor positive Lum A cancers, it is possible that BCL9 interacts with proteins other than β-catenin, and its activity may be, in part, independent of Wnt/β-catenin [93]. In this regard, it is interesting that BCL9 binds to proteins that transmit signals from estrogen receptor, thus connecting its overexpression to estrogen receptor-dependent transcriptional activity [80]. Moreover, in the case of invasive lobular carcinoma, it has been reported that a member of the Wnt protein family, WNT4, is transcriptionally induced by estrogen receptors and drives non-canonical Wnt signaling in lobular cancer cells [84]. Therefore, although pathway analysis connected BCL9 and PYGO2 protein to the “beta-catenin:TCF transactivating complex,” it is possible that those proteins play a special role in beta-catenin-independent signaling pathway both in ductal and lobular carcinomas.

The WWOX gene is located at 16q23.1–23.2, in a region containing the common fragile sites FRA16D, and its deletions have been observed in a large number of breast cancer cases [94]. In epithelial cells, WWOX, a WW-domain containing protein, modulates gene transcription through interaction with p73, AP-2gamma, and ERBB4 proteins [94,95]. Pathway analysis by the Reactome database pointed out the significant involvement of the WWOX gene, only in subgroup D2, in the pathway “Nuclear Signaling by ERBB4.” Such a pathway can be considered another example of the interaction between the 16q gene WWOX and the gamma-secretase complex subunits encoded on chr1q, since WWOX binds to a cytosolic fragment of the membrane receptor ERBB4, which is generated by the gamma secretase complex, and prevents its translocation to the nucleus [43]. Moreover, WWOX has been reported as an inhibitor of the Wnt pathways [96] by its interactions with the three members of the Dishevelled (Dvl) family. Therefore, the decreased expression of WWOX in 16q-loss cancers can contribute to the hyper-activation of Wnt pathways.

The use of curated knowledge-bases, such as Reactome, allows one to explore gene interactions with a certain degree of confidence in the experimental validation of functional pathways. However, the identification of novel interactions or the dissection of complex interactions can be difficult to attain by this methodology. Several interactions reported in the scientific literature are not necessarily revealed by this type of analysis. A first example is represented by the deubiquitinating enzyme CYLD, the familial cylindromatosis tumor suppressor gene, that acts as a negative regulator of proximal events in Wnt signaling at the level of the Dvl proteins, thus potentially having a role in both beta-catenin-dependent and -independent Wnt pathways [97]. Indeed, CYLD is located in chromosome 16q and its expression is reduced in 16q-loss cancer groups (A, B1, and D), thus suggesting that its decreased function could cooperate in the hyperactivation of conventional or non-conventional Wnt pathways. A second example is provided by the CBFB gene (Core-Binding Factor Subunit Beta), the beta subunit of a heterodimeric core-binding transcription factor that has been reported as frequently mutated in breast cancer [50]; it is located in chromosome 16q and shows a decreased expression in 16q-loss chromogroups (Figure 7B) and a high frequency of point mutations in group A and B1 (Figure S5). Indeed, a role for CBFB in the suppression of breast cancer has recently emerged, and it has been reported that nuclear CBFB/RUNX1 complex represses the oncogenic NOTCH signaling pathway in breast cancer [98]. Moreover, an efficient function of the CBFB/RUNX1 complex is necessary for the maintenance of the normal mammary epithelial phenotype [99]. In particular, the CBFB/RUNX1 complex represses NOTCH3 [98], and this observation establishes another interesting link among the underexpression of a 16q gene (CBFB)—the overexpression of 1q-located gamma-secretase component and the pathway “NOTCH3 Activation and Transmission of Signal to the Nucleus,” as described in previous paragraphs.

Finally, several functional interactions among the “core 1q-OverUpT” genes could be tracked to well-described molecular changes supporting cancer progression, such as SLC30A1 that encodes a zinc transporter [100], Trim46 that (together with 1q-located TRIM11 and TRIM17) belongs to a large gene family involved in breast cancer [101,102], and TUFT1 (Tuftelin) that promotes triple negative breast cancer metastasis and stemness by upregulating the Rac1/beta-catenin pathway [103].

Though this study provides an accurate description of breast cancers classified in five groups according to aberrations of chromosomes 1 and 16, its main limitation was that the hypothesis on the functional involvement of specific genes in the cancer pathogenesis must be experimentally verified in clearly defined models. The reported molecular characterization provides a fundamental guide in the generation and/or selection of such breast cancer models.

## 5. Conclusions

The integrated genomic analysis of 1,16-chromogroups provided the following insights on pathogenesis of invasive breast adenocarcinomas:

(1) Invasive lobular carcinomas could be observed both in the presence or in the absence of 16q-loss, although 16q-loss-associated lobular carcinomas were much more frequently observed (155/201, 77%), as already reported [45]. Interestingly, 16q-disomic lobular carcinomas were found to be a distinct subgroup of cancers characterized by a near-euploid karyotype, suggesting that a different form of genome instability is driving this cancer subtype.
(2) In the presence of a significant aneuploidy score (>4), 16q-loss was found to be a main determinant of lobular carcinomas, as shown by the lack or rarity of this phenotype in 16q-disomic groups B2 and C.
(3) The frequent co-occurrence of 1q-gain and 16q-loss could be observed in both ductal and lobular carcinomas, although a substantial proportion of lobular carcinomas (group D) could occur in the absence of 1q-gain.
(4) Transcriptome and pathway analysis revealed several dysregulated 1q- and 16q- genes that are overexpressed or underexpressed in 1,16-chromogroups in both ductal and lobular cancers and highlighted functional networks that may underlie the breast cancer progression. 1q-located genes, such as BCL9 and gamma-secretase components, might play central roles in such cooperating networks.

This analysis of 1,16-chromogroups provides essential information for the generation and selection of appropriate cancer cell models that recapitulate the molecular features observed in breast cancers bearing aberrations of chromosomes 1 and 16, and it generates a series of testable hypotheses on actionable functional pathways that can be investigated in such models.

## Figures and Tables

**Table 1:** Number of samples and arm-level copy number criteria for each 1,16-chromogroups. G: gain; L: loss; Dis: disomy; NF: no filter during selection; w/o: without; SNP: single nucleotide polymorphism; WES: whole exome sequencing. For more details, see Table S1.

Arm-Level Copy Number				1,16-Chromogroups	Brief Description of Copy Number Aberrations in chr1 and chr16	Cytogenetic Chromosome Aberration Inspiring Copy Number Criteria	Number of Samples		
1p	1q	16p	16q				SNP Array	RNA-Seq	WES
Dis	G	G/Dis	L	Group A	1q-gain and 16q-loss	der(1;16)	178	175	151
L	G	NF	NF	Group B	1q-gain/1p-loss	i(1q)	171	165	151
L	G	G/Dis	L	Subgroup B1	B with 16q-loss	i(1q)	101	98	98
L	G	Dis	Dis	Subgroup B2	B w/o 16q-loss	i(1q)	18	17	17
NF	G	Dis	Dis	Group C	1q-gain and normal chr16		90	89	85
Dis	Dis	G/Dis	L	Group D	16q-loss and normal chr1		75	72	69
Dis	Dis	Dis	L	Subgroup D1	D w/o 16p-gain	del(16q)	28	27	25
Dis	Dis	G	L	Subgroup D2	D with 16p-gain		47	45	44
Dis	Dis	Dis	Dis	Control (CTRL)	No aberrations in chr1 and chr16		71	68	48

**Table 2:** Number of 1q- and 16q-OverT and 1q and 16q-UnderT in 1,16-chromogroups.

1,16-Chromogroups	1q-OverT *	Shared with A	% of A	1q-UnderT **	Shared with A	% of A	OverT/UnderT Ratio
**A**	756	756	100	180	180	100	4.20
**B1**	737	634	83.86	180	130	72.22	4.09
**B2**	452	387	51.19	31	22	12.22	14.58
**C**	727	557	73.68	68	45	25.00	10.69
**D1**	34	29	3.84	64	50	27.78	0.53
**D2**	64	42	5.56	204	114	63.33	0.31
	16q-OverT *	**Shared with A**	**% of A**	16q-UnderT **	**Shared with A**	**% of A**	**OverT/UnderT Ratio**
**A**	20	20	2.65	418	418	100	0.05
**B1**	32	12	1.59	328	281	67.22	0.10
**B2**	55	7	0.93	22	19	4.55	2.50
**C**	60	10	1.32	48	40	9.57	1.25
**D1**	4	2	0.26	216	212	50.72	0.02
**D2**	10	5	0.66	354	301	72.01	0.03

* FCvsCTRL (linear fold-change (FC) between a selected 1,16-chromogroup and the CTRL group) > 1.3, adjp < 0.05; ** FCvsCTRL < −1.3, adjp < 0.05.

**Table 3:** Number of 1q- and 16q-UpT (Upregulated Transcripts) and 1q and 16q-DownT (Downregulated Transcripts) in 1,16-chromogroups.

1,16-Chromogroups	1q-UpT *	Shared with A	% of A	Shared with CTRL	% of CTRL	1q-DownT **	Shared with A	% of A	Shared with CTRL	% of CTRL	UpT/DownT Ratio
**A**	939	939	100	525	88.53	312	312	100	229	61.73	3.01
**B1**	897	840	89.46	526	88.70	298	259	83.01	218	58.76	3.01
**B2**	744	690	73.48	511	86.17	241	204	65.38	196	52.83	3.09
**C**	898	798	84.98	550	92.75	260	225	72.12	210	56.60	3.45
**D1**	487	476	50.69	426	71.84	373	261	83.65	277	74.66	1.31
**D2**	480	469	49.95	423	71.33	479	284	91.03	320	86.25	1.00
**CTRL**	593	525	55.91	593	100	371	229	73.40	371	100	1.60
	16q-UpT *	**Shared with A**	**% of A**	**Shared with CTRL**	**% of CTRL**	16q-DownT **	**Shared with A**	**% of A**	**Shared with CTRL**	**% of CTRL**	**UpT/DownT Ratio**
**A**	138	138	100	129	40.44	375	375	100	135	92.47	0.37
**B1**	179	126	91.30	162	50.78	320	300	80.00	135	92.47	0.56
**B2**	301	118	85.51	251	78.68	117	110	29.33	90	61.64	2.57
**C**	329	128	92.75	275	86.21	164	161	42.93	122	83.56	2.01
**D1**	104	89	64.49	102	31.97	290	280	74.67	129	88.36	0.36
**D2**	129	102	73.91	119	37.30	359	309	82.40	145	99.32	0.36
**CTRL**	319	129	93.48	319	100	146	135	36.00	146	100	2.18

* FCvsN > 1, adjp < 0.05; ** FCvsN < −1, adjp < 0.05.

**Table 4:** GSEA (gene set enrichment analysis) analysis of cytogenetic group A vs. CTRL (1058 samples; Analysis I). Reactome pathways with normalized enrichment score (NES) > 1.5.

Pathway Name	Size	ES	NES
* NRIF_SIGNALS_CELL_DEATH_FROM_THE_NUCLEUS	16	0.710	1.828
* NOTCH2_ACTIVATION_AND_TRANSMISSION_OF_SIGNAL_TO_THE_NUCLEUS	22	0.620	1.767
*° MITOCHONDRIAL_TRNA_AMINOACYLATION	18	0.776	1.754
*° SYNTHESIS_OF_GLYCOSYLPHOSPHATIDYLINOSITOL_GPI	18	0.736	1.746
*° MITOCHONDRIAL_FATTY_ACID_BETA_OXIDATION	36	0.556	1.634
*° TP53_REGULATES_TRANSCRIPTION_OF_GENES_INVOLVED_IN_CYTOCHROME_C_RELEASE	20	0.597	1.626
*° ENERGY_DEPENDENT_REGULATION_OF_MTOR_BY_LKB1_AMPK	29	0.588	1.610
* DEACTIVATION_OF_THE_BETA_CATENIN_TRANSACTIVATING_COMPLEX	42	0.552	1.608
* ZINC_TRANSPORTERS	17	0.582	1.599
*° FORMATION_OF_INCISION_COMPLEX_IN_GG_NER	43	0.595	1.587
*° INTRAFLAGELLAR_TRANSPORT	54	0.553	1.580
*° TRNA_AMINOACYLATION	24	0.667	1.576
*° SUMOYLATION_OF_DNA_METHYLATION_PROTEINS	16	0.663	1.569
*° CILIUM_ASSEMBLY	200	0.528	1.568
*° PEROXISOMAL_PROTEIN_IMPORT	63	0.518	1.543
*° ANCHORING_OF_THE_BASAL_BODY_TO_THE_PLASMA_MEMBRANE	96	0.545	1.542
° DISEASES_ASSOCIATED_WITH_N_GLYCOSYLATION_OF_PROTEINS	17	0.628	1.541
*° RESOLUTION_OF_ABASIC_SITES_AP_SITES	38	0.608	1.535
*° RAB_GERANYLGERANYLATION	65	0.488	1.528
*° RNA_POLYMERASE_III_TRANSCRIPTION_TERMINATION	23	0.589	1.528
* CELL_DEATH_SIGNALLING_VIA_NRAGE_NRIF_AND_NADE	76	0.497	1.528

* indicates pathways including 1q genes among the leading edge genes; ° indicates pathways including 16p genes among the leading edge genes.

**Table 5:** REACTOME pathways showing a normalized enrichment score (NES) > 1.48 in GSEA Analysis II.

Pathway Name	Size	ES	NES
NRIF_SIGNALS_CELL_DEATH_FROM_THE_NUCLEUS	16	0.70	1.74
TP53_REGULATES_TRANSCRIPTION_OF_GENES_INVOLVED_IN_CYTOCHROME_C_RELEASE	20	0.65	1.68
MITOCHONDRIAL_TRNA_AMINOACYLATION	18	0.77	1.67
DEFECTIVE_C1GALT1C1_CAUSES_TN_POLYAGGLUTINATION_SYNDROME_TNPS	17	0.69	1.66
DEACTIVATION_OF_THE_BETA_CATENIN_TRANSACTIVATING_COMPLEX	42	0.59	1.65
DISEASES_ASSOCIATED_WITH_N_GLYCOSYLATION_OF_PROTEINS	17	0.73	1.64
RNA_POLYMERASE_III_TRANSCRIPTION_TERMINATION	23	0.64	1.61
SYNTHESIS_OF_GLYCOSYLPHOSPHATIDYLINOSITOL_GPI	18	0.71	1.60
RNA_POLYMERASE_III_CHAIN_ELONGATION	18	0.67	1.57
NONSENSE_MEDIATED_DECAY_NMD	116	0.66	1.57
FORMATION_OF_THE_BETA_CATENIN_TCF_TRANSACTIVATING_COMPLEX	31	0.61	1.57
DEFECTIVE_GALNT3_CAUSES_FAMILIAL_HYPERPHOSPHATEMIC_TUMORAL_CALCINOSIS_HFTC	16	0.67	1.56
SELENOAMINO_ACID_METABOLISM	109	0.67	1.56
RESOLUTION_OF_ABASIC_SITES_AP_SITES	38	0.64	1.56
ENERGY_DEPENDENT_REGULATION_OF_MTOR_BY_LKB1_AMPK	29	0.60	1.55
GAP_FILLING_DNA_REPAIR_SYNTHESIS_AND_LIGATION_IN_GG_NER	25	0.63	1.51
SULFUR_AMINO_ACID_METABOLISM	28	0.54	1.51
EUKARYOTIC_TRANSLATION_ELONGATION	94	0.73	1.50
MITOCHONDRIAL_FATTY_ACID_BETA_OXIDATION	36	0.54	1.50
TRNA_AMINOACYLATION	24	0.66	1.49
PROLACTIN_RECEPTOR_SIGNALING	15	0.58	1.49
RESPONSE_OF_EIF2AK4_GCN2_TO_AMINO_ACID_DEFICIENCY	102	0.67	1.49
NOTCH2_ACTIVATION_AND_TRANSMISSION_OF_SIGNAL_TO_THE_NUCLEUS	22	0.56	1.49

## Data Availability

Publicly available datasets were analyzed in this study. This data can be found here: https://portal.gdc.cancer.gov (accessed on 29 October 2019).

## Supplementary Materials

The following are available online at https://www.mdpi.com/article/10.3390/cancers13071585/s1. Figure S1: schematic workflow of analysis performed in the study, Figure S2: The normalized chromosomal distribution index (NCDI) of 1q-OverT and 16q-UnderT calculated for each cytogenetic band of a single chromosome arm, Figure S3: Values of the “modified linear FCvsCTRL” for some representative genes belonging to cluster 1, 3, and 4, Figure S4: Oncoplot showing point mutations detected by WES in 645 samples out of 709 ductal and 149 lobular BRCA samples analyzed in TCGA study, Figure S5: Oncoplots showing point mutations detected by WES in 1,16-chromogroups, Table S1: Number samples of SNP-arrays RNAseq and WES analysis, Table S2: 1q-OverUpT in all 1,16-chromogroups, Table S3: 16q-UnderT in all 1,16-chromogroups, Table S4: Metascape_result, Table S5: Analysis I and II GSEA Real Values, Table S6: Analysis I and II GSEA ABS values, Table S7: LvsD DEGs.
