# Supplementary material for: Aberrations of Chromosomes 1 and 16 in Breast Cancer: A Framework for Cooperation of Transcriptionally Dysregulated Genes
Source: Cancers (Basel). 2021 Mar 30;13(7):1585. doi: 10.3390/cancers13071585 (PMC8037453; doi:10.3390/cancers13071585)
Supplement: Supplementary file 1 [file cancers-13-01585-s001.zip › cancers-1132998-supplementary materials/cancers-1132998-supplementary materials.pdf]

cancers MDPI

Supplementary Materials: Aberrations of Chromosomes 1 and 16 in Breast Cancer: A Framework for Cooperation of Transcriptionally Dysregulated Genes

Anna Provvidenza Privitera, Vincenza Barresi and Daniele Filippo Condorelli

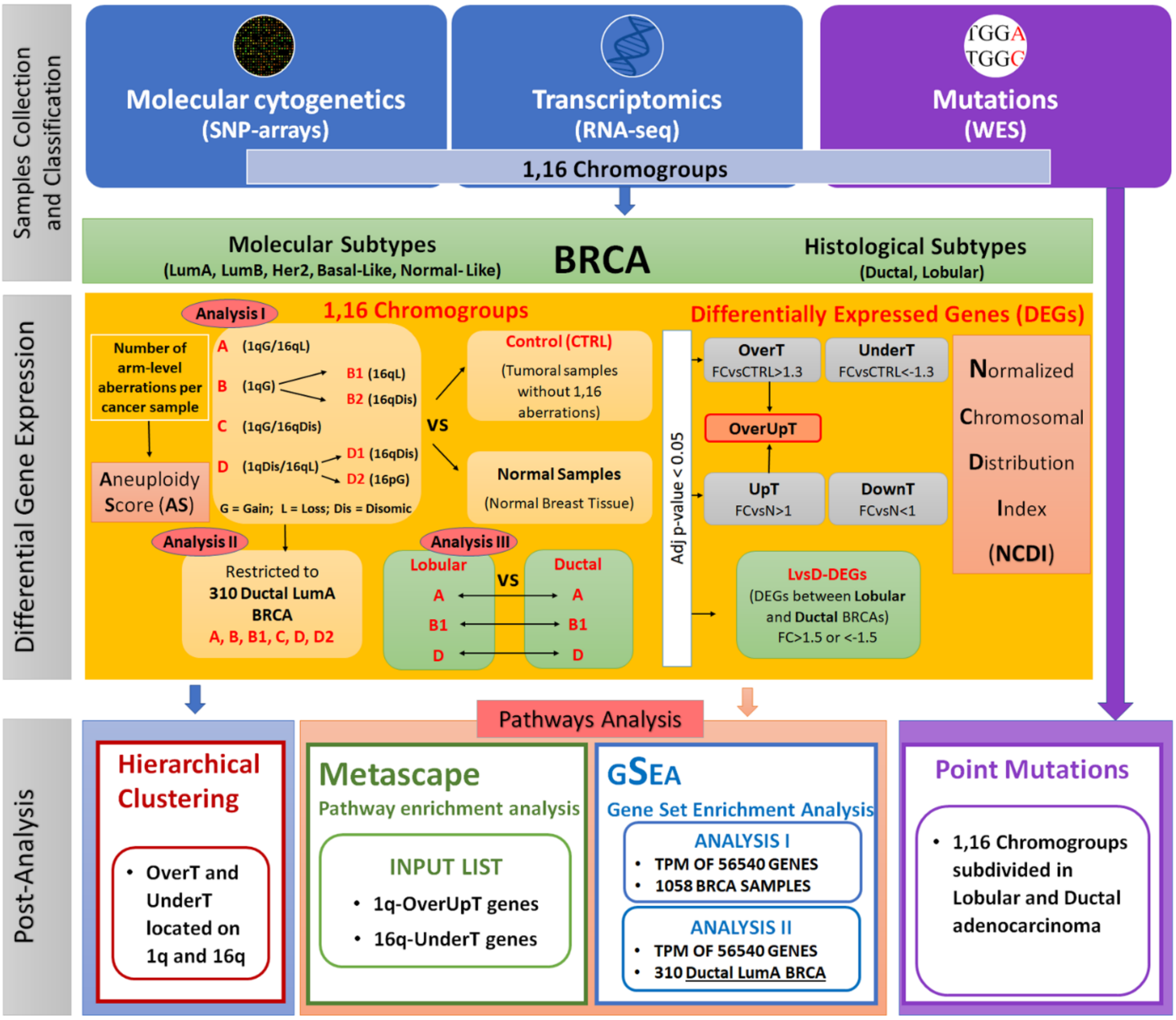

Figure S1. Schematic workflow of analysis performed in the study.

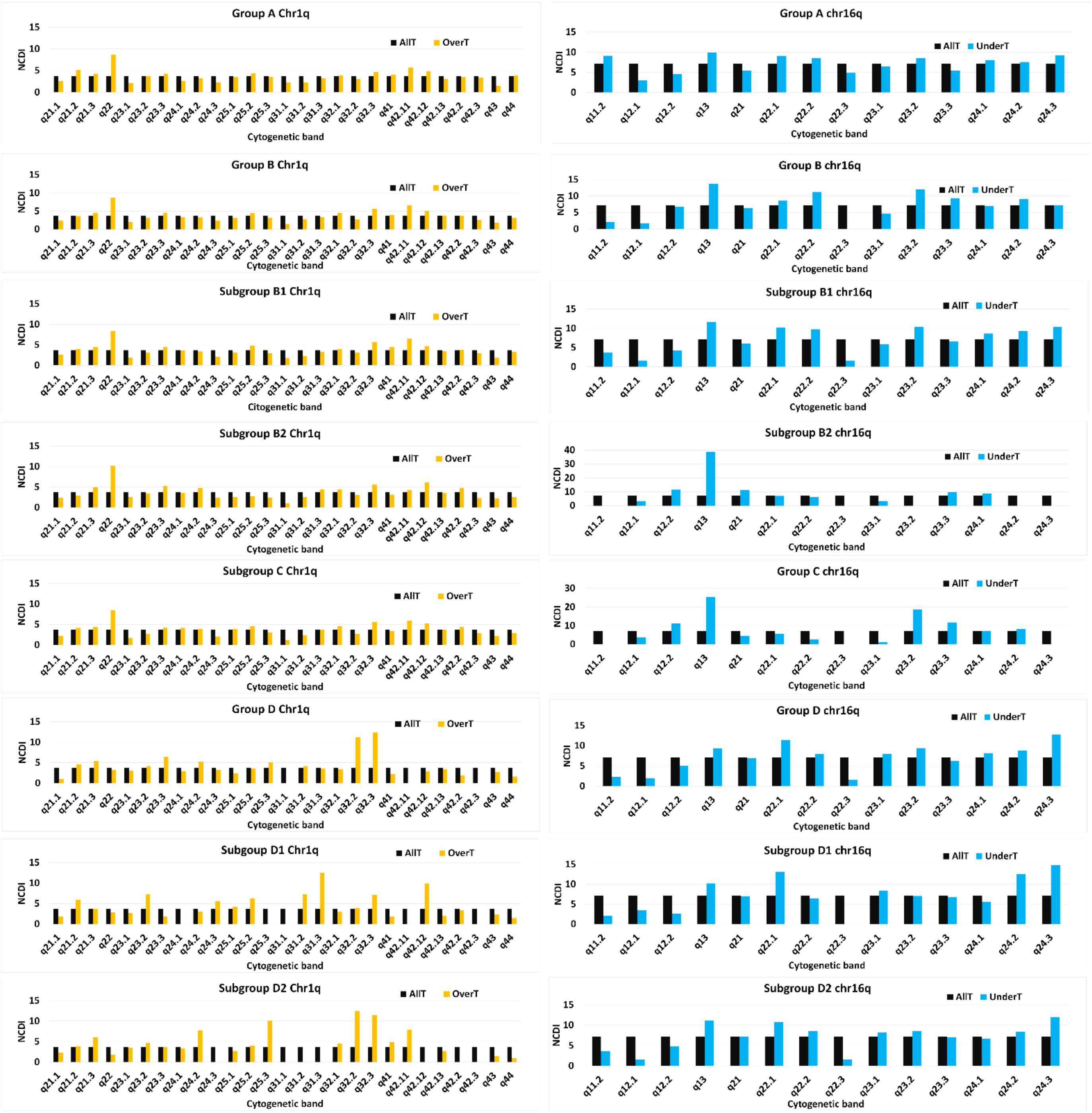

Figure S2. The Normalized Chromosomal Distribution Index (NCDI) of 1q-OverT (left graphs) and 16q-UnderT (right graphs) calculated for each cytogenetic band of a single chromosome arm (1q left, 16q right). NCDI values of all transcripts (AllT) analyzed by RNA-seq in each cytogenetic band are also reported for comparison.

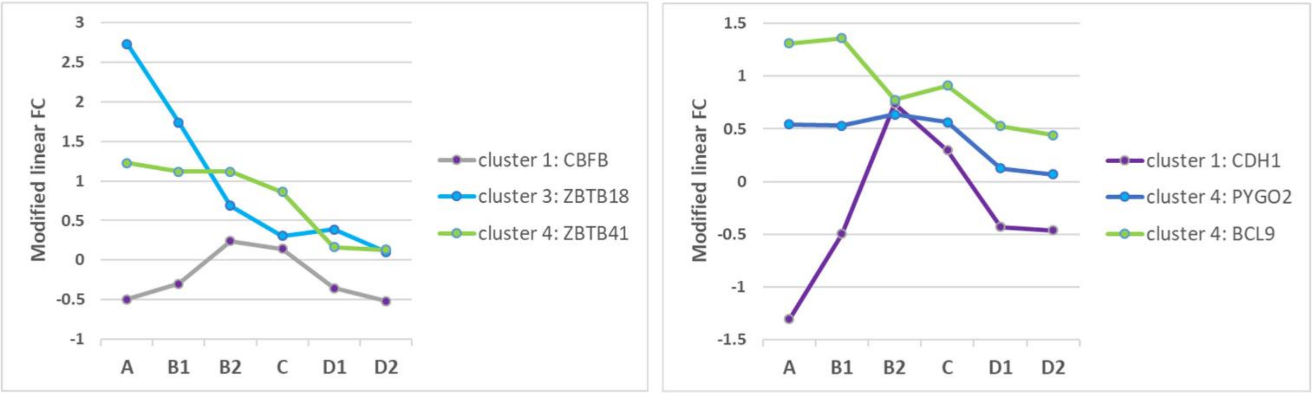

Figure S3. Values of the "modified linear FCvsCTRL" for some representative genes belonging to cluster 1, 3 and 4.

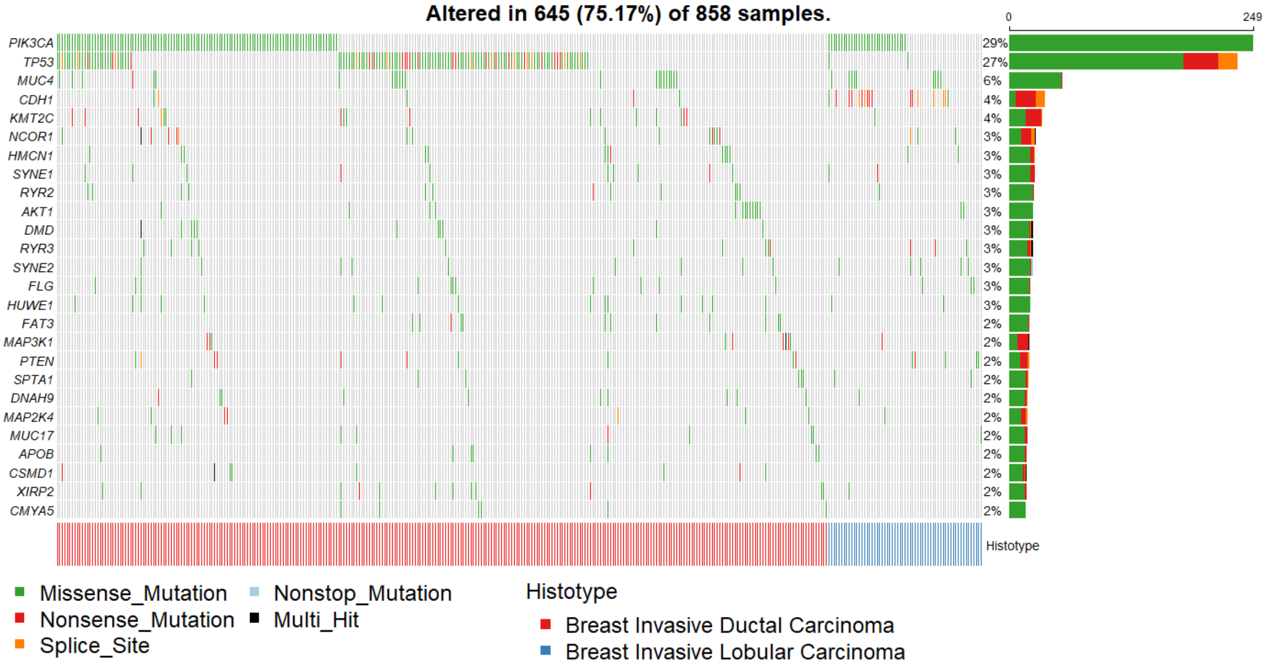

Figure S4. Oncoplot showing point mutations detected by WES in 645 samples out of 709 ductal and 149 lobular BRCA samples analysed in TCGA study.

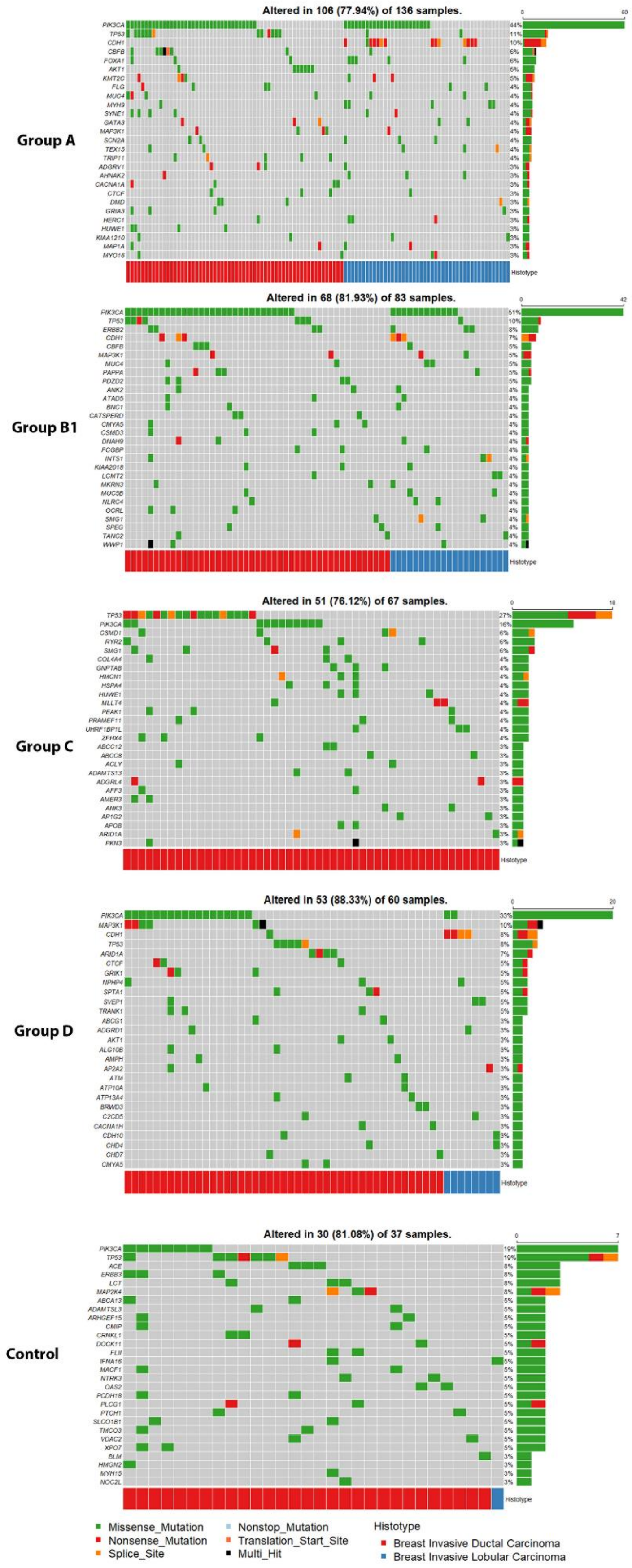

Figure S5. Oncoplots showing point mutations detected by WES in 1,16-chromogroups.
